# Genetically engineered mesenchymal stem cells with dopamine synthesis for Parkinson’s disease in animal models

**DOI:** 10.1038/s41531-022-00440-6

**Published:** 2022-12-22

**Authors:** Jun Li, Nan Li, Jingkuan Wei, Chun Feng, Yanying Chen, Tingwei Chen, Zongyong Ai, Xiaoqing Zhu, Weizhi Ji, Tianqing Li

**Affiliations:** 1 State Key Laboratory of Primate Biomedical Research, Institute of Primate Translational Medicine, Kunming University of Science and Technology, 650500 Kunming, Yunnan China; 2 Yunnan Key Laboratory of Primate Biomedical Research, 650500 Kunming, Yunnan China

**Keywords:** Parkinson's disease, Parkinson's disease, Parkinson's disease

## Abstract

Although striatal delivery of three critical genes for dopamine synthesis by viruses is a potential clinical approach for treating Parkinson’s disease (PD), the approach makes it difficult to finely control dopamine secretion amounts and brings safety concerns. Here, we generate genetically engineered mesenchymal stem cells encoding three critical genes for dopamine synthesis (DOPA-MSCs). DOPA-MSCs retain their MSC identity and stable ability to secrete dopamine during passaging. Following transplantation, DOPA-MSCs reinstate striatal dopamine levels and correct motor function in PD rats. Importantly, after grafting into the caudate and putamen, DOPA-MSCs provide homotopic reconstruction of midbrain dopamine pathways by restoring striatal dopamine levels, and safely and long-term (up to 51 months) correct motor disorders and nonmotor deficits in acute and chronic PD rhesus monkey models of PD even with advanced PD symptoms. The long-term benefits and safety results support the idea that the development of dopamine-synthesized engineered cell transplantation is an important strategy for treating PD.

## Introduction

Increasing dopamine concentrations in the striatum are the most effective therapeutic strategy for Parkinson’s disease (PD). The standard treatment is the administration of pharmacological agents, such as oral administration of the dopamine precursor 3,4-dihydroxy-l-phenylalanine (l-DOPA), which is converted to dopamine upon crossing the blood‒brain barrier into the brain to transiently increase concentrations of brain dopamine and thereby discontinuously modulate neuronal activity in the striatum, the primary target of dopaminergic neurons. Supplementation with l-DOPA alleviates motor symptoms, but systemic delivery stimulates all of the dopaminergic systems, including the brain, resulting in off-target effects, and long-term treatment can be thought to cause drug-related side effects, such as dyskinesias, cognitive impairment, and off-target nonmotor symptoms^1–3^. Furthermore, l-DOPA can only be converted into dopamine in the action of the aromatic amino acid decarboxylase (AADC) enzyme, which is lost in advanced PD patients. Recently, a more continuous and local dopamine replacement strategy was developed by attempting to target the synthesis and release of l-DOPA or dopamine to the striatum using gene therapy vectors engineered to express key enzymes in the dopamine synthesis pathway^1,4–6^.

**Fig. 1:**
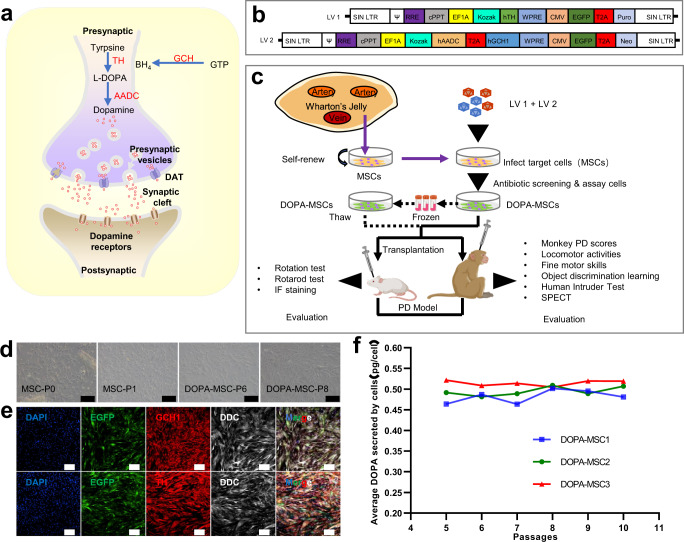
Establishment of DOPA-MSCs that can stably secrete dopamine. **a** Dopamine biosynthetic route. AADC, TH, and GCH1 are three critical enzymes involved in dopamine biosynthesis. DAT dopamine transporter. Adapted from ref. ^78^. DAT element obtain Licensing Rights from BioRender.com. **b** Schematic diagram showing two vector structures of the lentiviruses carrying human *AADC*, *TH,* and *GCH1*. **c** A schematic of the experimental process. Apart from elements created by our co-authors, other elements were created with BioRender.com. **d** Morphological characteristics of MSCs (P0, P1) and DOPA-MSCs (P6, P8). Scale bars: 100 μm. **e** TH staining of DOPA-MSCs showing coexpression of EGFP, TH, DDC, and GCH1. Scale bars: 200 μm. TH tyrosine hydroxylase, DDC dopa decarboxylase, GCH1 GTP-cyclohydrolase 1. **f** Secretion of dopamine DOPA-MSCs during passaging assayed by a dopamine ELISA Kit. Three independent cell lines were examined.

Dopamine biosynthesis is driven by three key enzymes: tyrosine hydroxylase (TH), catalyzing the synthesis of l-DOPA from the amino acid tyrosine; AADC, converting l-DOPA to dopamine; and GTP-cyclohydrolase 1 (GCH1) as the rate-limiting step in the production of the TH enzyme cofactor tetrahydrobiopterin^1,7,8^ (Fig. 1a). ProSavin, a lentiviral vector creating dopamine “factories” in nondopaminergic striatal neurons of the sensorimotor putamen by delivering the genes encoding three key enzymes in the dopamine biosynthesis pathway, was evaluated in preclinical and clinical studies^4–6,9^. Injection of a tricistronic lentiviral vector encoding the three genes into the striatum in the PD macaque monkey safely restored extracellular concentrations of dopamine and corrected the motor deficits for 12 months without associated dyskinesias^5^. ProSavin was previously shown to be well tolerated in phase I/II first-in-human study, with significant improvements in motor behavior within 5 years and even 8 years of follow-up^9^. However, the clinical data indicate that dopamine replacement may require further optimization^1,9^. Recently, OXB-102, an optimized gene expression cassette to increase DA production, has been shown to achieve a significantly higher dopamine yield than ProSavin^10^ and is currently in an active phase 1/2 safety and dose escalation study (AXOLenti-PD study, NCT03720418). These studies showed that gene therapy based on the three key enzymes may be an attractive and feasible strategy to cure PD patients. Despite these successes, direct delivery of the three genes into the striatum by viral infection still faces many obstacles due to the presence of drug-related adverse events. One important limiting factor is that it is difficult to finely evaluate and control which cells or how many cells in the striatum could be successfully infected by delivered viruses, leaving us not to finely control the amount of the desired transgene expression for synthesizing dopamine. The second concern is the safety of gene therapy caused by viruses as a delivery tool, including lentiviral and AAV viruses^11,12^. A recent report showed that some of the gene fragments carried by the AAV virus are integrated into the dog’s chromosomes, and 44% of the integrations are near genes involved in cell growth, raising important safety concerns^13^. In the clinic, three children with a rare neuromuscular disease died after directly receiving a high dose of gene therapy with the AAV virus in a clinical trial run by Audentes Therapeutics (https://www.biopharmadive.com/news/audentes-gene-therapy-patient-deaths/580670/,2020), further raising safety concerns. However, these limitations may be resolved by developing genetically engineered cells to allow the continuous and stable synthesis of dopamine followed by cell transplantation^14^. Since the safety and characteristics of these cells could be fully evaluated before transplantation, the strategy can therefore provide a robust and reproducible “off the shelf” cell source for treating PD.

Mesenchymal stem cells (MSCs) provide an effortless cell source with a low incidence of immune rejection and tumorigenesis response and few adverse effects and therapy-related complications^15^. The easy availability, low immunogenicity and immunomodulatory abilities of MSCs render these sources most practical for experimental and possible clinical applications by auto and allogeneic transplantation. Previous studies revealed that inflammation is related to PD, including astrogliosis and microgliosis^16,17^. The modulatory properties of MSCs have been extensively studied^18^. Human MSCs have neuroprotective effects on dopaminergic neurons through anti-inflammatory action^19,20^. MSC treatment has been reported to inhibit the transmission of α-synuclein and protect neurons from apoptosis in a Parkinsonian model^21,22^. These results showed that MSCs have some functions in delaying PD progression by preserving the nigrostriatal pathway. To fully combine the unique characteristics of MSCs and gene therapy, we introduced the three genes *TH*, *AADC,* and *GCH1* into human umbilical cord-derived MSCs (MSCs) and obtained MSCs with the stable ability to synthesize dopamine (DOPA-MSCs). Due to the complicated pathogenesis and significant interindividual variability in clinical PD patients, one animal model alone does not well reflect the broad effectiveness of cell transplantation. Therefore, we used a PD rat^23^ model as well as PD monkey models induced by three different methods according to previous reports and our developed methods in our primate facility^24–26^, namely, acute PD models by administration of MPP+ into the putamen (Putamen MPP) or internal carotid delivery of MPTP (Carotid MPTP), and chronic PD models by lateral ventricle delivery of MPP+ (Bi-ventricle MPP+). Our results showed that DOPA-MSC grafts in the striatum can safely correct motor deficits and cognitive impairment over the long term in PD rats and monkeys by restoring extracellular concentrations of dopamine, providing proof-of-concept for their use in a wider range of PD patients.

## Results

### Establishment of DOPA-MSCs that can stably secrete dopamine neurotransmitter

To obtain DOPA-MSCs, we isolated MSCs from Wharton’s jelly of human umbilical cords and infected MSCs with two lentiviruses carrying *TH*, and *AADC* and *GCH1*, respectively (Fig. 1b, c and Supplementary Fig. 1). After antibiotic selection, DOPA-MSCs coexpressing the three genes were obtained (Fig. 1d). The expression of TH, GCH1, and DDC protein was further confirmed by immunostaining (Fig. 1e). Based on analysis of three different cell lines, the average content of dopamine secretion was ~0.5179 ± 0.0522 pg per DOPA-MSC and remained over expansion (Fig. 1f), indicating that DOPA-MSCs stably synthesize dopamine neurotransmitter.

We next assessed whether lentivirus infection affects MSC identities and found that DOPA-MSCs maintained multiple differentiation abilities and expressed surface marker proteins of MSCs (Supplementary Fig. 2a, b). We also examined the gene expression profiles of DOPA-MSCs and MSCs. The analysis of differentially expressed genes (DEGs) between them showed that the numbers of upregulated and downregulated DEGs in DOPA-MSCs were 181 and 171, respectively (Supplementary Fig. 2c). KEGG pathway and GO term analyses indicated that DEGs are not related to MSC identities. The downregulated genes in DOPA-MSCs were enriched with amino acid transmembrane transport; organic acid transport; glycine, serine, and threonine metabolism; and biosynthesis of amino acids, whereas the upregulated genes were related to virus responses and immune and dopamine biosynthetic process (Supplementary Fig. 2d, e). Therefore, DOPA-MSCs reserved MSC identities.

### Safety of DOPA-MSCs

Since cells infected by lentivirus may exhibit insertional mutagenesis^27^, which would raise safety concerns, we analyzed cancer-related TP53 mutations in three DOPA-MSC lines by RNA-seq data according to a well-established protocol^28,29^ and did not detect any reported mutation sites of TP53 in these DOPA-MSCs (Supplementary Fig. 3a). By adopting the expressed-SNP-karyotyping (eSNP-karyotyping) method for global gene expression analysis^30^, we further evaluated chromosomal aberrations and analyzed loss of heterozygosity (LOH) and did not detect any chromosomal aberrations or LOH events in these DOPA-MSCs (Supplementary Fig. 3b, c).

To assess the safety of DOPA-MSCs in vivo, we transplanted DOPA-MSCs into the striatum of NOD.CB17-Prkdc^scid^/NcrCrl mice with immunodeficiency. Cell survival and well-defined grafts were observed at the site of injection in the striatum 5 months postinjection (Supplementary Fig. 4a). No tumorigenicity, ectopic proliferation, or neuronal abnormalities were detected in the brain (Supplementary Fig. 4a, b). Transplanted cells elicited minimal astrocyte glial scarring but did not cause host Iba1^+^ microglia to gather around graft cores (Supplementary Fig. 4a). Histopathological assessments of major organs showed no evidence of adverse proliferation, tumorigenicity, ectopic tissue formation, or other serious safety issues related to the transplanted cells in all animals on Day 150 post-injection (Supplementary Fig. 4b). Together, these data showed that the lentivirus infections and overexpression of *TH*, *AADC*, and *GCH1* did not raise any additional adverse effects or safety concerns.

### DOPA-MSC grafts rescue motor deficits in parkinsonian rats

**Fig. 2:**
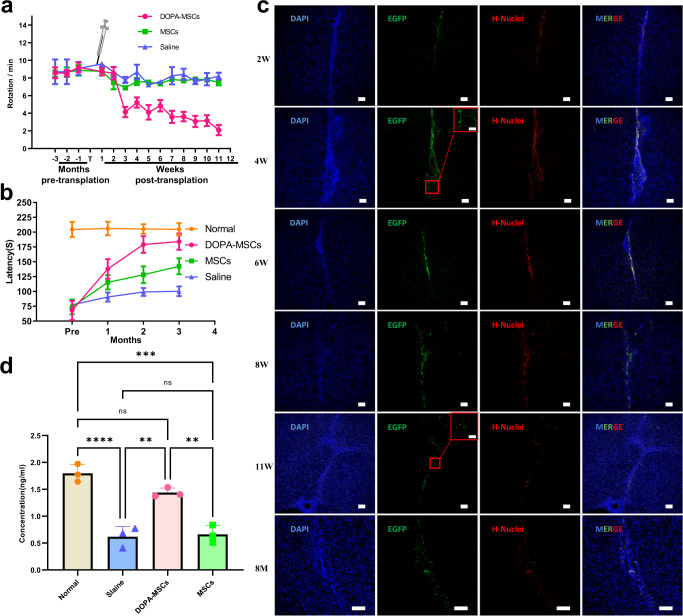
DOPA-MSCs can survive long-term and reverse functional deficits in PD rats after transplantation. **a** and **b** Behavioral analysis. **a** Apomorphine-induced rotation analysis in the three groups grafted with DOPA-MSCs (*n* = 13), MSCs (*n* = 3), or PBS (*n* = 3). **b** The rotarod test showing the changes in latency time to fall in PD rats before and after transplantation. The results are expressed as the time (second) that rats remained on an accelerating rotarod before falling. Data are presented as the mean ± SEM. In the two behavioral tests, Two-way ANOVA was followed by Holm–Sidak test. **c** Immunofluorescence staining for grafts placed in the striatum of 6-OHDA lesioned rats after 1, 2, 4, 6, 7, 11 weeks, and 8 months. EGFP^+^, H-Nuclei^+^. Double-staining of EGFP^+^ and H-Nuclei^+^ indicated that these cells were derived from grafted DOPA-MSCs. Scale bars: 100, 50 μm in the zoom panel. H-Nuclei, human nuclei; EGFP, Enhanced Green Fluorescent Protein. **d** Dopamine concentration in cerebrospinal fluid from three different treated rats 3 months after transplantation. Data are presented as the mean ± SEM. One-way AONVA analysis of variance was used to compare the differences between groups, and significant differences were evaluated by the Bonferroni test (**p < 0.01, ***p < 0.001).

To assess whether DOPA-MSCs can be used to rescue PD phenotypes as expected, we transplanted DOPA-MSCs into the stratum of unilateral 6-hydroxydopamine (6-OHDA)-induced adult PD rats. The wild-type MSCs as a control were also transplanted into the stratum of PD rats. Before transplantation, all 6-OHDA-induced PD rats used for cell grafts exhibited 7.97 ± 0.78–8.70 ± 1.40 rotations per minute and stabilized the phenotypes for at least 3 months. The effects of DOPA-MSC transplantation were examined in PD animals by quantification of rotations induced by apomorphine^31^ each week after transplantation. Neither rats that received MSC grafts nor PBS-treated rats in the dopamine-denervated striatum showed obvious improvement in apomorphine-induced rotations (*p* > 0.05) (Fig. 2a). In contrast, apomorphine-induced rotations were significantly (*p* < 0.05) and quickly improved in all PD rats that received DOPA-MSCs within 3 weeks after cell grafts (Fig. 2a). Furthermore, these improved phenotypes were retained until sacrifice. To further assess the improvement of PD symptoms, we used the rotarod assay to examine motor coordination and balance^32^ at 3 months postgraft. The latency time to fall for DOPA-MSC grafted PD rats was significantly (*p* < 0.01) extended compared with those of MSC- or PBS-treated PD rats (Fig. 2b). Histological analysis showed that DOPA-MSC survival was preserved for at least 8 months in the PD striatum by examining the coexpression of EGFP and human nuclei protein (Fig. 2c). Contrary to the results in immunodeficient mice (Supplementary Fig. 4a), the transplanted cells elicited the gathering of host Iba1^+^ microglia around the graft core and induced glial scar formation, showing that immune rejection may still occur after transplantation of DOPA-MSCs into PD rat brains without immunosuppression (Supplementary Fig. 5a), although it is unclear whether the possible immune response is caused by xenogeneic cell transplantation. Furthermore, CD45, a marker of leukocyte, immunostaining showed that CD45^+^ cells were absent in grafts, indicating no leukocyte infiltration or adaptive immunity (Supplementary Fig. 5b).

To evaluate whether the phenotypic improvements of PD rats grafted with DOPA-MSCs benefited from dopamine transmitter secretion, we analyzed dopamine concentrations in cerebrospinal fluid (CSF) 3 months postgraft. As expected, the dopamine amounts in rats receiving DOPA-MSCs were significantly (*p* < 0.05) higher than those in the PBS-treated rats and were comparable to (*p* > 0.05) those in wild-type rats (Fig. 2d). Together, these results suggested that grafts of DOPA-MSCs in the striatum alleviated motor dysfunction in PD rats by secreting dopamine transmitters.

### DOPA-MSC grafts improve behaviors and increase DA concentrations in monkeys with acute PD

Monkeys can suffer from PD naturally^33^. The unique motor skills, working memory and neuroanatomical complexity of nonhuman primates closely resemble those of humans^34^, providing a context to understand the pathophysiology of PD and accurate models to assess the safety and efficiency of cell or gene therapy in treating human diseases, especially over the long term. We, therefore, established PD rhesus monkeys. Age is an important factor that could affect PD pathogenesis^11^ and the therapeutic efficacy in PD patients who receive fetal tissue grafts^35^. Given that younger monkeys often spontaneously recover over time after MPTP treatments^36–39^, we chose 8–15-year-old rhesus macaques (Supplementary Table 1) to generate stable PD models and assessed the animals’ parkinsonism using a clinical PD score, spontaneous locomotor activity, and a fine-motor skill (FMS). Given that PD pathogenesis is very complex, the use of only one animal model does not well reflect the broad effectiveness of cell transplantation. Therefore, we used three methods to induce PD monkey models according to previous reports and our developed methods in our primate facility^24–26^, including acute PD models by administration of MPP+ into the putamen (Putamen MPP) or internal carotid delivery of MPTP (Carotid MPTP), and chronic PD models by lateral ventricle delivery of MPP+ (Bi-ventricle MPP+).

**Fig. 3:**
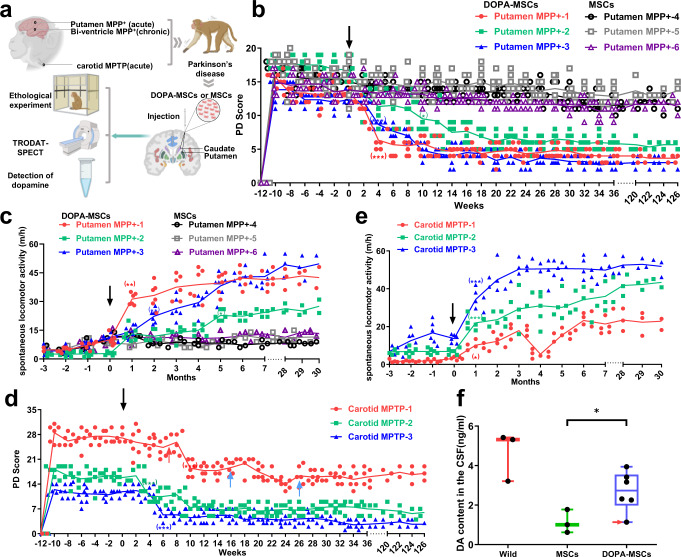
DOPA-MSC transplantation reverses functional deficits in acute hemiparkinsonian monkeys. **a** A schematic of DOPA-MSC therapy in PD monkeys. This figure was created with BioRender.com. **b** Changes in PD scores from 12 weeks before to 36 weeks after DOPA-MSCs (*n* = 3) or MSCs (*n* = 3) were transplanted into putamen-lesioned PD monkeys. The values are the average of multiple blinded evaluations (*n* = 6). A two-way analysis of variance (ANOVA) with Dunnett’s multiple comparisons test was performed. *p < 0.05; **p < 0.01; ***p < 0.001; **c** Changes in spontaneous locomotor activity from 3 months before transplantation to 30 months after transplantation of putamen-lesioned monkeys receiving DOPA-MSC (*n* = 3) or MSC (*n* = 3) transplantation. The value is the mean of 6 tests per month (*n* = 6). A two-way analysis of variance (ANOVA) with Dunnett’s multiple comparison test was performed. *p < 0.05; **p < 0.01; ***p < 0.001; **d** Changes in PD scores of MPTP-induced parkinsonism monkeys (*n* = 3) receiving DOPA-MSCs from 3 months before transplantation to 30 months after transplantation. The values are the mean of multiple blinded evaluations (*n* = 6). Significance before and after transplantation was assessed by a two-tailed Student’s *t*-test. The red and blue arrows indicate the second transplantation and intravenous injection of DOPA-MSCs for the Carotid MPTP-1 monkey, respectively. **e** Changes in spontaneous locomotor activity from 3 months before transplantation to 30 months after transplantation of MPTP-induced parkinsonism (*n* = 3) receiving DOPA-MSC transplantation. Significance before and after cell transplantation was assessed by a two-tailed Student’s *t*-test. The red color represents the Carotid MPTP-1 monkey, the green color represents the Carotid MPTP-2 monkey, and the blue color represents the Carotid MPTP-3 monkey, respectively. **f** Quantification analysis of dopamine concentration in the cerebrospinal fluid of DOPA-MSC-treated monkeys (*n* = 6), MSC-grafted monkeys (*n* = 3), and wild monkeys (*n* = 3). Data are presented as the mean ± s.d. Significance was assessed by a two-tailed Student’s *t*-test. The red arrow indicates the data of Carotid MPTP-1 monkeys.

Since axons of dopamine neurons target the putamen, direct delivery of MPP+ or 6-hydroxydopamine into the rat striatum results in degeneration of the nigral dopamine neurons^25,26^. We therefore directly injected MPP+ into the putamen to induce PD in monkeys (Fig. 3a). As expected, blinded evaluations showed that after a single injection of MPP+ into the putamen of the striatum, all the monkeys (*n* = 6) quickly displayed typical PD symptoms within 1–2 weeks, including bradykinesia, postural and gait imbalances, and slight tremors and impairments in gross motor skills in the hand (Fig. 3b). After PD symptoms persisted over the 3-month period, 6 × 10^6^ DOPA-MSCs or wild-type MSCs were precisely transplanted into the bilateral putamen and caudate in these PD monkeys, respectively. Given that MSCs have low immunogenicity, no immunosuppressants were used.

Unblinding of the collected data over 31 months (126 weeks) following transplantation revealed that the animals with DOPA-MSC grafts (*n* = 3) showed a sustained and significant recovery (*p* < 0.05) beginning from the 2nd week, whereas monkeys receiving wild-type MSCs (*n* = 3) remained unchanged (Fig. 3b). The Putamen MPP+-1, -2 and -3 monkeys that received DOPA-MSCs began to improve 2 weeks after transplantation; exhibited significant (*p* < 0.05) recovery in defense, balance, posture, bradykinesia, gross motor skills, tremor and gait at the 4th, 10th and 4th weeks, respectively; and were stable for at least 30 months, with an average PD score recovery ratio of 70% and reaching as high as 90% (the Putamen MPP+-3 monkeys) (Fig. 3b and Supplementary Fig. 6a). The three monkeys, but not wild-type MSC-grafted monkeys, exhibited significant improvement in spontaneous locomotor activity and could use their four limbs to walk at the 1st (the Putamen MPP+-1 monkey), 5th (the Putamen MPP+-2 monkey) and 1st month (the Putamen MPP+-3 monkey) (Fig. 3c). The hunched posture of the three monkeys was almost completely recovered at the 18th (Putamen MPP+-1), 26th (Putamen MPP+-2) and 18th (Putamen MPP+-3) weeks. The climbing ability of the damaged contralateral upper limbs in the cage, which is difficult for PD monkeys, was greatly improved (Video S1). This quick recovery of PD symptoms is high in contrast to a gradual onset (approximately 6–8 months) of functional motor improvement in PD monkeys after transplantation of dopamine neurons derived from pluripotent stem cells^37–39^.

Given that PD monkey models in most studies are induced by unilateral right intracarotid artery (ICA) infusion of the neurotoxin MPTP^40^, we also created a stable unilateral PD model by this method. Monthly blinded evaluations showed that the monkeys (*n* = 3) developed bradykinesia, postural and gait imbalances, as well as slight tremors and impairments in gross motor skills in the contralateral (left) hand (Fig. 3d). However, the symptoms of the three monkeys exhibited marked individual differences. The Carotid MPTP-1 and -2 monkeys often sat in the cage with a decrease in movement and exhibited defense response deficits, poker faces, sluggishness, drooling disorder, and poor balance (Fig. 3d and Supplementary Fig. 6b). The Carotid MPTP-3 monkey’s symptoms were mild, but typical symptoms were observed, including bradykinesia, tremor, and gait imbalance (Fig. 3d). The Carotid MPTP-1 monkey exhibited the most serious symptoms, including serious tremor, and its PD score (~26.7) was close to the highest score (30) that can be rated by the PD score (Fig. 3d). The monkey almost completely lost the ability to stand up, eat, autonomously move and grasp, and it required artificial nursing, including infusion and feeding.

Before transplantation, these PD phenotypes were stabilized for three months (Fig. 3d). After DOPA-MSC transplantation, the Carotid MPTP-2 and -3 monkeys began to exhibit improved PD phenotypes within 2–3 weeks, exhibited significant (*p* < 0.05) recovery at the 4th or 6th week, and stabilized for at least 30 months, with an average PD score recovery ratio of approximately 60% (the Carotid MPTP-2 monkey) and 70% (the Carotid MPTP-3 monkey), respectively (Fig. 3d). These significantly improved phenotypes included defense response, gait, posture, bradykinesia, tremor and spontaneous locomotor activity (Fig. 3d, e, Supplementary Fig. 6b and Video S2). After the first transplantation, we found that the therapeutic effect on the Carotid MPTP-1 monkey was not ideal, but it is worth noting that the monkeys’ tremor symptoms were relieved, it could eat by itself and began to move autonomously (*p* = 0.0142) (Fig. 3d and Supplementary Fig. 6b). Although its gait was unbalanced, it could move slowly. Seven weeks later, we performed a second transplantation with 6 × 10^6^ DOPA-MSCs. Four weeks after transplantation, the muscle rigidity of the monkey was relieved, the hind limbs could start to walk in strides, and the movement speed was markedly (*p* < 0.001) improved. The monkey could climb but was still unable to move freely and often fell down in the cage (Fig. 3d, e, and Supplementary Fig. 6b). Unfortunately, the recovery only lasted for approximately 3 weeks. Some PD symptoms relapsed again, resulting in difficulty standing up. Due to poor health status, anesthesia for putamen transplantation is risky. According to previous results in our primate facility, PD monkeys with such advanced symptoms are very prone to death, especially when denervated gastrointestinal dopamine neurons damage the self-feeding ability of monkeys. To resume the circuit of gastrointestinal dopamine neurons, we, therefore, performed an intravenous injection of DOPA-MSCs with a cell number of 1 × 10^7^. After transplantation, the monkey’s symptoms, including overall spontaneous activity and posture, gradually and significantly improved, and the defense response basically returned to a normal state (Fig. 3d, e and Supplementary Fig. 6b), implying that grafted DOPA-MSCs may promote appetite in PD monkeys by functioning in the gastrointestinal system. After 10 weeks, we performed a second intravenous injection of DOPA-MSCs. The relieved symptoms were maintained until the 30th month (Fig. 3d, e).

To address whether the PD symptom recovery resulted from the increase of dopamine transmitter secreted by grafted DOPA-MSCs, we assessed the content of dopamine in the cerebrospinal fluid (CSF) of these 6 acute PD monkeys that received DOPA-MSCs (three putamen MPP+ and three carotid MPTP monkeys), three MSC-treated monkeys and three wild-type (Wild) monkeys. The results showed that the dopamine concentration in the CSF of MSC-treated monkeys was 1.136 ± 0.587 ng/ml. In contrast, the dopamine concentration in the DOPA-MSC-grafted monkeys was significantly (*p* = 0.0443) increased up to 3.941 ± 1.006 ng/ml (close to 4.651 ± 1.247 ng/ml in wild monkeys; Fig. 3f). In agreement with no ideal benefit of DOPA-MSCs on the Carotid MPTP-1 monkey, the dopamine concentration in the CSF of monkey was not significantly restored (Fig. 3f). These data demonstrated that DOPA-MSCs ameliorated PD symptoms in monkeys by secreting dopamine transmitters.

### DOPA-MSC grafts long-term alleviated motor behaviors in chronically induced PD monkeys

**Fig. 4:**
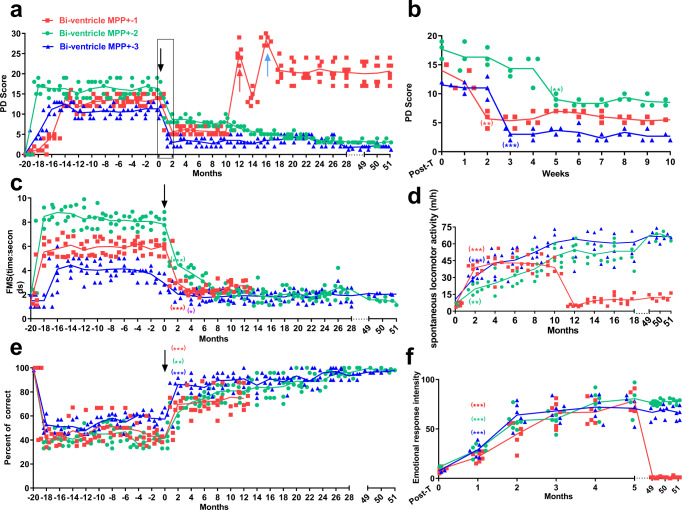
DOPA-MSC grafts rescued the behaviors of bilateral chronic PD monkeys induced by lateral ventricle administration of MPP^+^. **a** and **b** Changes in PD scores of chronic PD monkeys receiving DOPA-MSCs (*n* = 3) from 20 months before transplantation to 51 months after transplantation. Each value is from the average result of 6 blind evaluations. The red and blue arrows indicate the second transplantation and intravenous injection of DOPA-MSCs for the Bi-ventricle MPP+-1 monkey, respectively. **b** Changes in monkey PD scores during the indicated times for the indicated times in the red box in panel (**a**). **c** FMS showing changes in single grasping time in PD monkeys (*n* = 3) before and after receiving DOPA-MSCs. Each value is based on the average of 4 experiments before transplantation and of 6 experiments posttransplantation. **d** Primate SCAN showing changes in spontaneous locomotor activity after DOPA-MSC transplantation. Each value is from the average result of 6 tests per month. **e** The working memory tests showing the correct rate of PD monkeys before and after DOPA-MSC transplantation. Each value is from the average result of 6 tests. **f** Emotional response assays showing the improvement of emotional responses in PD monkeys after transplantation. Each value is from the average result of six tests. Two-tailed Student’s *t*-test was performed by self-comparisons of the monkeys before and after tests; *p < 0.05; **p < 0.01; ***p < 0.001.

Given that PD of most patients is chronically and progressively occurred in the clinic, we next sought to develop a chronic monkey model of PD for simulating clinical patients. Our previous studies showed that the lateral ventricle MPP+ administration model can induce monkeys to develop chronic PD by low-dose MPP+ administration twice a week^24,41^. The administration period lasted for 3 months and progressively generated chronic PD monkey models (Supplementary Fig. 7a). After MPP+ administration ended, the Bi-ventricle MPP+-1 monkey’s symptoms successively exhibited transient deterioration, slight recovery after 3 months and stabilization (Fig. 4a). The main symptoms included sluggishness, poker faces, the rigidity of right limb muscles, movement difficulty, and imbalanced posture (Supplementary Fig. 7b–d). The Bi-ventricle MPP+-2 monkey exhibited more advanced symptoms with little spontaneous recovery, which were stabilized within 2 months after the first MPP+ administration until cell transplantation (Fig. 4a). These symptoms included lethargy, tremor, muscle rigidity of four limbs, severe motor impairment and imbalance when standing or walking, dependence on cages for support, defensive response deficits and progressive deterioration, even after the cessation of MPP+ treatment (Fig. 4a). The Bi-ventricle MPP+-3 monkey’s symptoms were similar to those of the Bi-ventricle MPP+-2 monkey, but its symptoms were slightly milder.

Before transplantation, these monkeys’ PD symptoms were stably maintained for 1.5 years to exclude the possibility of spontaneous recovery during the treatment (Fig. 4a). A total of 6 × 10^6^ DOPA-MSCs were transplanted into the four sites of the bilateral putamen and caudate nucleus (one site/region). The PD symptoms of the three monkeys after transplantation were largely improved within 2, 3, and 5 weeks (Fig. 4a, b). With the recovery of motor function, self-feeding and FMS ability (*p* = 0.0046) were also significantly improved (Fig. 4c, Video S3). The Bi-ventricle MPP+-2 monkey, which was unable to stand up before transplantation, was able to climb up to the top of the cage with hands 5 weeks after transplantation (Video S4). Although the limb muscles were still rigid, the gross motor skill (*p* = 0.0012) and spontaneous locomotor activity (*p* = 0.0071) were significantly increased (Fig. 4d and Supplementary Fig. 7c). The defensive response, crawling in the cage, movement, and balance ability were largely improved (Supplementary Fig. 7c). Nevertheless, the monkey still exhibited a slight back bend, and its gait did not return to the normal level. Three weeks after transplantation, the Bi-ventricle MPP+-3 monkey exhibited a marked decrease (*p* < 0.001) in the PD score and tremor symptoms; restoration of the defense response; improvements in gross motor skills, grasping, hand and hindlimb movement and gait (Fig. 4b and Supplementary Fig. 7d); and spontaneous locomotor activity (Fig. 4d, *p* < 0.001). These improved PD symptoms were maintained until at least the 51st month after transplantation (Fig. 4a).

Two weeks after transplantation, the Bi-ventricle MPP+-1 monkey’s PD phenotypes were significantly relieved (*p* = 0.0033), including movement disorder, defense response, bradykinesia, gross motor skills, and balance (Fig. 4b and Supplementary Fig. 7b). There was a significant improvement in FMS (*p* = 0.0018) and spontaneous locomotor activity (*p* < 0.001) (Fig. 4c, d). The right limb could be used to walk. Unfortunately, the PD symptoms of the monkey recurred 11 months after grafting. Muscle rigidity, stooped back and poor balance in both hindlimbs reappeared on both sides. Its balance ability and hindlimb rigidity were slightly improved when the l-DOPA drug was administered. However, after continuous administration for 3 days, the monkey no longer reacted to the drug l-DOPA. We therefore immediately performed another putamen transplantation of DOPA-MSCs. Three weeks later, the monkey’s symptoms were significantly improved again, including reduced leg stiffness, walking on the legs, and restoring postural balance. However, after only 11 months of this treatment, the PD symptoms recurred again and became more severe. The monkey completely lost autonomous movement and the ability to stand up and eat. We therefore intravenously injected 1 × 10^7^ DOPA-MSCs as performed in the Carotid MPTP-1 monkey, once a week, for a total of 6 times. It is worth mentioning that after every transplantation, the monkey’s appetite and mental state were significantly improved. After the fifth vein transplantation, the monkey could stand up and resume self-feeding and move independently, but its other typical PD symptoms were not significantly alleviated (Fig. 4a). The state was maintained until at least the 51st month.

### DOPA-MSC transplants long-term alleviated nonmotor symptoms in chronically induced PD monkeys

Nonmotor symptoms are also important symptoms in the pathogenesis of PD, such as cognitive function impairments with slower learning rates or retaining tasks^42^, which cannot be treated effectively by levodopa administration or high-frequency deep brain stimulation (DBS) targeting the STN region^43^. The cognitive deficits in PD patients are associated with making more random errors or guesses^44^. We, therefore, conducted an object discrimination learning experiment to assess the changes in the monkeys’ cognitive ability (see the “Methods” section). Our results showed that the success rates of the three PD monkeys in finding the correct target at one time were 46%, 67%, and 56%, respectively. Dramatic improvements (Bi-ventricle MPP+-1 monkey, *p* < 0.001; -2 monkey, *p* = 0.0018; -3 monkey, *p* < 0.001) in cognitive deficits were observed 2 months after grafting and even reached ~90% of the normal state at the later stage (Fig. 4e and Video S5).

In addition, PD patients commonly experience depression and anxiety^45^, which have received limited attention. We found that the three Bi-ventricle MPP+ monkeys had symptoms such as lethargy, sluggishness and poker faces before transplantation. Therefore, the alternation of defensive movements and emotional performance in the three monkeys before and after transplantation can more intuitively reflect their spiritual changes. We found that although the symptoms of the three monkeys were quite different, the improvement in emotional response was very significant 2 months after transplantation (*p* < 0.001) and was continuously increased (Fig. 4f and Video S6). Together, the results demonstrate that DOPA-MSC grafts can rescue nonmotor phenotypes in PD monkeys.

### Transplanted DOPA-MSCs improve PD symptoms by secreting dopamine transmitters

**Fig. 5:**
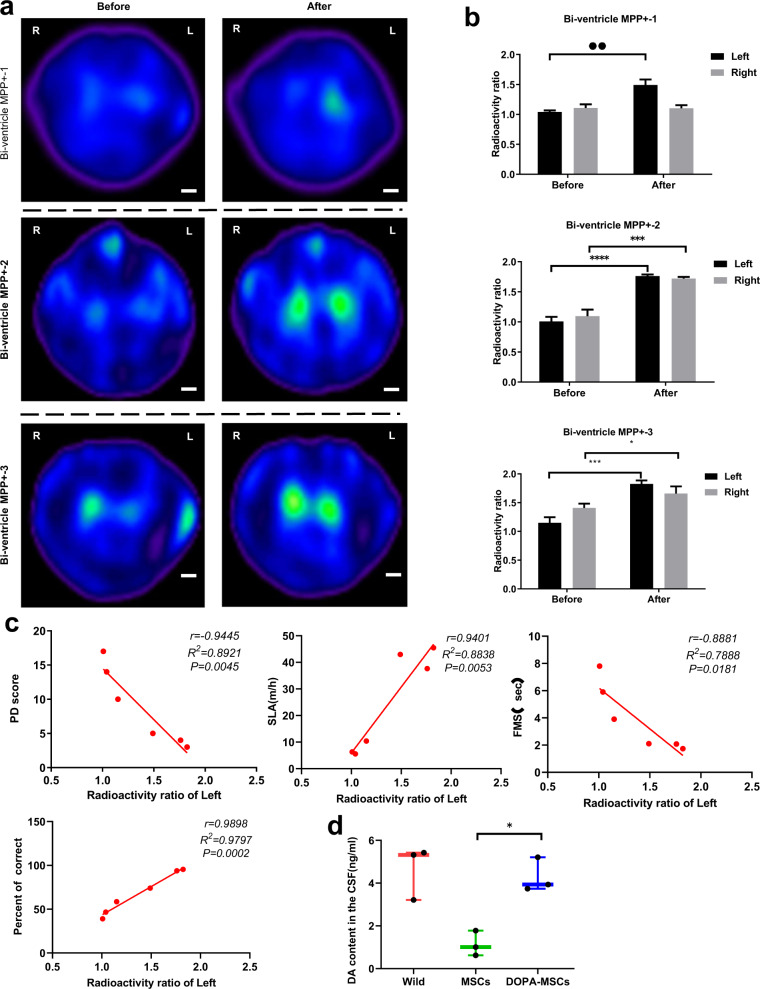
DOPA-MSCs improved PD symptoms by secreting dopamine transmitters. **a** Radioactive uptake images of the position of the basal ganglia before and 6 months after DOPA-MSC transplantation in chronic PD monkeys (*n* = 3). Scale bars, 1 cm. **b** Quantification of the radiation ratios of the left and right basal ganglia before and after DOPA-MSC transplantation in chronic PD monkeys. Data are presented as the mean ± s.d. (*n* = 3). Significance was assessed by a two-tailed Student’s *t*-test. *p < 0.05; **p < 0.01; ***p < 0.001. **c** Correlation between the ratio of radioactive uptake in the left basal ganglia and behavioral results in the Bi-ventricle MPP+ group. Two-tailed Pearson’s correlation analysis was performed, and *r* and *P* values are shown. SLA spontaneous locomotor activity. **d** Detection of dopamine concentrations in cerebrospinal fluid from different treatment monkeys. Data are presented as the mean ± s.d. (*n* = 3). Significance was assessed by a two-tailed Student’s *t*-test, *p < 0.05; **p < 0.01; ***p < 0.001.

To further evaluate DOPA-MSC therapy for the PD monkeys objectively, we performed ^99^Tc^m^-TRODAT-1 SPECT imaging detection. Dopamine transporter (DAT) is a glycoprotein located in the presynaptic membrane of dopamine neurons, which has been demonstrated to have a close relationship with striatal dopamine levels^46,47^. Studies have shown that using ^99^Tc^m^-TRODAT-1 as an imaging agent combined with single-photon emission computed tomography (SPECT) can be used to evaluate dopamine activity in PD patients^48^ or monkeys^49^. Therefore, we used the combination of ^99^Tc^m^-TRODAT-1 SPECT brain dopamine transporter imaging and computerized region of interest (ROI) technology and semiquantitatively analyzed DAT function in the monkey bilateral striatum before and after transplantation by using the uptake ratio of ST (striatum)/CB (cerebellum) radioactivity as an indicator. Before transplantation and 6 months after transplantation, SPECT scans were performed on three monkeys that received lateral ventricle administration of MPP+. The results showed that the three monkeys exhibited weak radioactivity in both basal ganglia before transplantation (Bi-ventricle MPP+-1 monkey: left, *R* = 1.041 ± 0.026 and right, *R* = 1.106 ± 0.061; -2 monkey: left, *R* = 1.008 ± 0.075 and right, *R* = 1.096 ± 0.107; -3 monkey: left, *R* = 1.151 ± 0.096 and right *R* = 1.408 ± 0.075) (Fig. 5a, b). After grafting, the radioactivity uptakes of the Bi-ventricle MPP+-2 and -3 monkeys were concentrated in the bilateral striatum, with clear outlines of the striatum on both sides, and were almost evenly and symmetrically distributed throughout the striatal region on both sides (Fig. 5a). Their radioactivities were significantly increased after compared with pre-transplantation (Bi-ventricle MPP+-2 monkey: left *R* = 1.761 ± 0.026, *P* < 0.0001 and right *R* = 1.720 ± 0.031, *P* = 0.0006; -3 monkey: left *R* = 1.824 ± 0.062, *P* = 0.0005; right *R* = 1.659 ± 0.124, *P* = 0.0404). For the Bi-ventricle MPP+-1 monkey, only the left striatum showed significant accumulation of radioactivity (*L* = 1.491 ± 0.089, *P* = 0.0011) and not the right side (*R* = 1.102 ± 0.052, *P* = 0.9272) (Fig. 5b), which may partially explain why the therapeutic effect of DOPA-MSCs on this monkey is suboptimal.

To assess whether PD symptom alleviation results from dopamine secretion, we analyzed the correlation between the radioactivity of the left basal ganglia and the behavioral results. Linear regression results showed that radioactivity had a significant negative correlation with motor behavioral PD score (*r* = −0.9445, *P* = 0.0045) and FMS (*r* = −0.8881, *P* = 0.0181) and a significant positive correlation with spontaneous locomotor activity (*r* = 0.9404, *P* = 0.0053) and working memory (*r* = 0.9898, *P* = 0.0002) (Fig. 5c). Finally, we also assessed dopamine concentrations in the cerebrospinal fluids of monkeys 6 months after transplantation. We found that the average concentration of dopamine was 4.292 ± 0.798 ng/ml, which was significantly (*P* = 0.005) higher than that in MSC-treated monkeys and comparable to that in wild-type monkeys (Fig. 5d). Together, these results showed that PD symptom improvement results from dopamine secretion by DOPA-MSCs.

## Discussion

To date, a great diversity of approaches to treat PD have been evaluated due to the disease’s complexity. Strategies have been aimed at increasing dopamine levels, improving neuronal survival and preventing damage in the mitochondria by gene therapy, and replacing lost dopamine neurons using stem cell engraftment^50^. Gene therapy is an attractive approach to impart a durable effect on neuronal function through the introduction of genetic material to reestablish dopamine levels and/or functionally recover dopaminergic signaling through the restoration of dopaminergic activity. Ongoing clinical gene therapy trials in PD patients are focused on the enzymatic enhancement of dopamine production and/or the restoration of the nigrostriatal pathway to improve dopaminergic network function by virus delivery. However, post hoc graft volumetric analyses and postmortem autopsy data highlighted the need for higher virus titers and infusion volumes to achieve optimal putaminal coverage. Difficulties in precisely controlling the amount of the desired transgene expression, the potential oncogenicity, or other unanticipated consequences of insertional mutagenesis have limited its more widespread clinical use. In this study, to solve these shortcomings, we developed a new strategy to treat PD by continuous striatal delivery of dopamine through genetically engineered MSCs (DOPA-MSCs). The safety and characteristics of the engineered cells have widely been evaluated before transplantation. Importantly, the strategy could efficiently correct motor and cognitive deficits in PD rats and monkeys. The results up to 51 months demonstrated the long-term efficacy, feasibility, and safety of DOPA-MSCs in nonhuman primate models, implying that it is a potential strategy for clinical therapeutic applications for PD patients.

The idea of repairing the brain by replacing the neurons that die in PD has been a long-standing dream for researchers in the field^51^. Over the past few decades, many cell types have been proposed as candidates to replace dying neurons, including cells derived from the human fetal midbrain and dopaminergic progenitors/neurons differentiated from human pluripotent stem cells (PSCs)^52^. Transplantation of these cell types is being tested in the clinic^53^; however, limitations remain, including low rates of survival and integration into the host neural circuit, requirements of behavioral correction for a long time, and mixed and variable benefits of the transplanted neurons^54^. For example, the outcomes from fetal mesencephalic tissues are mixed and associated with dyskinesia, primarily due to undefined and unstandardized donor tissues^55,56^. In addition, after dopaminergic neuron precursors were transplanted into the monkey, <53% or 1% of cells in the graft survived in the brain^37,38,57^, even in PD monkeys that had received autologous transplantation from iPS-derived dopamine neurons^39,58^. Furthermore, before functioning in vivo, transplanted dopamine neurons or progenitors are required to differentiate into mature dopamine neurons and establish a functional circuit with host neurons. This is why some motor functional improvements could not be observed until 6 months posttransplantation^37,39,58^. These limitations suggest that motor functional improvements are due to fluctuations in behavioral performance. Therefore, the outcomes of cell transplantation become complicated in the clinic. In this study, we used a different strategy to quickly restore local dopamine concentrations in the caudate and putamen regions of PD monkeys by DOPA-MSC transplantation. As predicted, these PD model monkeys exhibited dramatic behavioral improvements within 2–3 weeks after transplantation. Importantly, most PD monkeys (7/9) exhibited long-term benefits with one surgery, providing a cost-effective or “off the shelf” treatment. We also found that nonmotor symptoms, including cognitive impairments and emotional disorders, were also significantly alleviated through DOPA-MSC transplantation, in agreement with the results obtained from neural precursor or autologous iPSC-derived dopamine neuron grafts in two recent studies^39,57^.

It is worth noting that, in contrast to mild or slight improvement over 6 weeks^57^ or 6 months after transplantation of differentiated DA neurons or progenitor cells into the striatum^37,39,58^, our transplantation strategy has a more rapid onset and yields significant therapeutic effects within one month (4 weeks), which clearly demonstrates that alleviation of PD symptoms benefits from DOPA-MSC grafts. This feature is undoubtedly an important advantage for severely ill PD patients. Previous studies have shown that advanced nigrostriatal lesions are detrimental to the survival of grafted cells due to the toxic local environment caused by lesions, resulting in poor recovery^57,59,60^. Consistent with these results, we also found that there are relationships between cell engraft outcomes and disease states in PD monkeys. This finding reflects the large interindividual variability in the lesion degree, engraftment efficiency and PD symptom expression. The Carotid MPTP-1 and recurred Bi-ventricle MPP + -1 monkeys exhibited very severe PD symptoms, and their overall motor function and self-feeding ability were almost completely lost. According to our previous results and experiences in our primate facility, such monkeys easily die due to nursing difficulties. Treating monkeys with such severe symptoms is a huge challenge. Although a minor recovery or clear absence of recovery occurred following grafting, even after a second transplantation and infusions, significant advancements were achieved, as the two monkeys with advanced PD symptoms no longer required manual care and could self-feed, and their PD symptoms were obviously relieved over the long term after DOPA-MSC transplantation. Interestingly, we found that the intravenous infusion of DOPA-MSCs could significantly relieve dyskinesia and appetite of PD monkeys, although it could not dramatically alleviate some PD symptoms. In addition, the continuous multiple transplants also demonstrated the safety of our DOPA-MSCs. In contrast, DOPA-MSC grafts achieve consistent and remarkable therapeutic effects in monkeys with PD scores below 20. In summary, these results showed that DOPA-MSCs may provide important clinical benefits for PD patients, including patients with advanced disease.

Although it is difficult to understand the reason why the two PD monkeys experienced relapse or even become worse once relapsed after transplantation, the fact that their nigrostriatal lesions may be too advanced, thus causing the relapse of PD monkeys, seems more likely due to immune rejection. Although MSCs and the brain have been reported to possess certain immune privileges, we still observed microglial aggregation around injected cells in rat brains, suggestive of possible immune rejection. However, one DOPA-MSC graft achieved functional effects over years in monkeys, implying that even if there is immune rejection, it is quite slow. Furthermore, the slow immune rejection may result from the transplantation of xenogeneic cells, as reported for allogenic transplantation in nonimmunosuppressed monkeys^61^. In the future, we can reveal the relationship of immune rejection with relapse and further elucidate the therapeutic mechanisms of DOPAM-MSCs in PD monkeys by postmortem immunohistological analysis, detection of neural circuits, and transcriptome analysis, including bulk and single-cell RNA-Seq.

Nonhuman primates are optimal models to develop procedures of cell therapy for clinical applications, including determining an optimal graft site and transplantation method of cells^62^. Interestingly, our preliminary experiments revealed that when DOPA-MSCs were only transplanted into the dorsal putamen of PD monkeys, the therapeutic effect was not obvious, and the monkeys exhibited vomiting symptoms (data not shown). A large number of PD patients suffer from dysfunction of the dopamine circuit in the caudate in addition to the putamen^63,64^. We, therefore, hypothesized that both the caudate and putamen as graft sites are necessary. As expected, the cotransplantation of DOPA-MSCs into both the caudate and putamen regions greatly increased the function of DOPA-MSCs. The phenomenon of vomiting may be due to the concentration of a large number of cells at a single site, which causes the brain area to be squeezed. Therefore, we subsequently performed multiple regions and site transplantations by selecting two sites of the putamen and one side of the caudate nucleus. By procedural optimization, we demonstrated that the grafted sites and methods are important factors that affect the therapeutic effects of DOPA-MSCs in PD. In contrast to cells, viruses using traditional stereotactic methods are subject to multiple sources of off-target delivery. These include reflux up the cannula, unintentional spread through perivascular spaces and misplacement of infusion cannulas^65^; such events occur even in the hands of very experienced and skilled surgeons. For cell transplantation, the delivery considerations appear to be less complicated. Unlike viral vectors, transplanted cells do not need to be distributed over a large area during surgical delivery to potentially produce a therapeutic effect. Cells that survive after transplantation sprout neurites well beyond the localized cell deposits^65^. In this study, the reason why multiple regions and site transplantation is so effective is that dopamine neuron axons project to a wide range of striata, and when transplanted cells are injected into one location, it is difficult for these transplanted cells to migrate outward through the striatum, resulting in ineffective diffusion of the secreted dopamine to a wide area. In addition, when the new circuits away from transplanted sites are depleted with dopamine, it will be difficult to maintain the therapeutic effects over time. Therefore, as in our present study, multiple-site transplantation is an effective solution to solve this problem. However, for too many site transplantations, surgery may cause additional brain trauma. Another alternative method is to inject other genes related to cell migration in combination with DOPA-MSC grafts, which may induce DOPA-MSC migration into wider regions and eventually result in better outcomes. Therefore, in the future, the usage of nonhuman primates to further optimize cell therapy procedures is crucial before clinical applications of cells.

The clinical pathogenesis of PD patients is highly diverse. Furthermore, there are substantial variabilities among patients in the clinical pathology of PD^66^. Therefore, the use of only one animal model does not fully reflect the wide-range effectiveness of cell transplantation. To better simulate the clinical applications to the greatest extent, we selected older monkeys and used three methods to generate PD monkey models according to previous reports and our developed methods in our primate facility^24–26^, namely, acute PD models by administration of MPP+ into the putamen (Putamen MPP) or internal carotid delivery of MPTP (Carotid MPTP) and chronic PD models by lateral ventricle delivery of MPP+ (Bi-ventricle MPP+). Furthermore, before transplantation, we reserved sufficient time to stabilize symptoms (3 months for acute models and 18 months for chronic models) to exclude the possibility of spontaneous recovery, allowing us to clearly observe the improvements in various PD symptoms benefiting from cell transplantation. The gross motor skills and loss of fine motor movements of the hand, which are the most common symptoms of PD patients, were remarkably alleviated. We also noted that some symptoms after DOPA-MSC transplantation, such as gait and curved back, were still not fully restored in some PD monkeys. These may be “sequelae” caused by muscle atrophy due to the muscle rigidity caused by long-term PD pathology before transplantation. Our systematic analysis also revealed a linear relationship between the radioactivity intensity in SPECT brain imaging and motor recovery. In addition, dopamine detection assays showed that the recovery of PD symptoms is concomitant with the restoration of dopamine concentrations in the brain. Together, our results strongly supported the significant therapeutic benefits of DOPA-MSC transplantation in both acute and chronic PD models. Together, our results provide a proof-of-concept for a wider range of PD patients.

In conclusion, the present findings provide interindividuality of disease progression and recovery, as well as a proof of concept preclinical study to demonstrate that grafts of genetically engineered MSCs with dopamine synthesis can significantly improve motor and nonmotor function in a nonhuman primate model of PD over a long period of time (at least 51 months). Our results will contribute to the development of translational medical techniques that use genetically engineered stem cells to treat intractable PD diseases. In the future, it is essential to determine the optimal and genetically engineered cells using site-directed insertion to avoid the use of viruses that can survive better after transplantation with good safety and are able to consistently produce dopamine and to continue to refine the clinical protocol for treating PD patients using nonhuman primates.

## Methods

### Isolation and culture of mesenchymal stem cells from Wharton’s jelly

The MSC isolation is ethically approved by the Medical Ethics Committee of Kunming University of Science and Technology. Fresh umbilical cord samples were obtained from normal full-term delivery mothers with written informed consent and reserved in a sterilized 4 °C phosphate-buffered saline (PBS) solution. After disinfection in 75% ethanol for 1 min, the umbilical cord vessels were cleared off and washed five times with PBS solution. The mesenchymal tissue (in Wharton’s jelly) was then diced into cubes of approximately 1–3 mm^3^. Tissue blocks were diluted into about 0.3–0.5 g/ml by using a self-made serum-free medium (SFM), and then inoculated on Petri dishes in the SFM after mixing by pipetting. The above dishes were placed in an incubator at 37 °C, 5% CO_2_, and saturated humidity. When the cell confluence rate reaches 80–90%, cells were digested with Tryple (Sigma) into single cells. The MSCs were then used directly for routine passaging or stored in liquid nitrogen for later use.

### Generation of DOPA-MSCs

Human CDS sequences of TH, ADDC and GCH1 were cloned into two plasmids, respectively. The plasmids pLV [Exp]-EGFP:T2A:Puro-EF1A>TH [NM_000360.3], pLV [Exp]-EGFP:T2A:Neo-EF1A > ADDC [NM_001082971.1]:T2A: GCHI [NM_001024024.1] were constructed. The plasmids vector maps are provided in Supplementary Fig. 1. Lentiviruses were generated in 293T cells by transfecting packaging and backbone plasmids using the calcium phosphate/DNA coprecipitation method. 293T cells were cultured in Dulbecco’s MEM (DMEM) containing 10% FBS. The supernatant containing the viral particles was collected 72 h after transfection and concentrated by ultracentrifugation at 27,000 rpm for 2 h at −4 °C. The viral particles were then resuspended in DMEM.

MSCs were cultured for 24 h when passaged at 2 × 10^4^ cells per 35 mm dish. The two concentrated lentiviruses including *TH*, *ADDC*, *GCH1* were mixed and added into the culture of MSCs in the presence of polybrene (5–10 µg/mL) for 12 h. On the 2nd day after infection, MSCs were harvested by Tryple digestion and were replated at 1 × 10^6^ cells per 100 mm dish. Seventy-two hours after infection, cells were co-treated with 300 μg/ml of G418 and 0.15 μg/ml puro for at least 14 days. The survival cells were routinely passaged according to MSC culture protocol. These stable MSCs were named DOPA-MSCs. DOPA-MSCs were harvested by Tryple digestion and cell pellets were resuspended at a cell density of 2 million cells/ml of Cryopreservation Solution in cryotubes before being placed in a controlled rate freezer (Thermo Fisher) to cryopreserve cell product. Amounts of dopamine secretion in the DOPA-MSCs were assayed by dopamine ELISA Kit (Abnova, Catalog Number KA1887).

### Flow cytometric analysis

Analysis of cells was performed using the human MSC Analysis Kit (BD biosciences, 562245) according to the manufacturer’s instructions.

### Trilineage differentiation

Osteogenic, adipogenic, and chondrogenic differentiation experiments were performed following the instructions of the human mesenchymal stem cell functional identification kit (OriCell^®^ HUXXC-90021, HUXXC-90031, HUXXC-90041). For osteogenic differentiation, 2 × 10^5^ cells were seeded per well in 6-well plates. When cells reached 50–70% confluency, the medium was replaced with an osteogenic differentiation medium and kept for 4 weeks. To assess osteogenic differentiation, immunofluorescence Alizarin Red S (OriCell^®^, ALIR-10001) staining was performed for the calcium-rich extracellular matrix. For adipogenic differentiation, cells were seeded into a 6-well plate at the density of 2 × 10^5^ cells/well, and maintained in a culture medium until 100% confluency. Then, cells were cultured in an adipogenic differentiation medium for 4 weeks. Lipid droplets of the resultant differentiated cells were detected using Oil red O (OriCell^®^, OILR-10001) staining. For chondrogenic differentiation, 3 × 10^5^ cells resuspended in chondrogenic differentiation medium were centrifuged for 3 min at 150 × g in 2-mL cryogenic vials (Corning, NY, USA). Then, cells were cultured for 4 weeks. After 4 weeks, the chondrogenic pellet was harvested and fixed in 4% paraformaldehyde (Sigma, STBJ9683). Cryosectioning was performed by Cryostat (Leica CM1950, Germany) and 5 µm sections were stained with Alcian Blue (OriCell^®^, ALCB-10001) staining.

### Transcriptome analysis

Total RNA was isolated from MSCs and DOPA-MSCs cultured in the SFM using the TRIzol™ Reagent (Thermo Fisher Scientific, 15596018). RNA sequencing libraries were constructed using the NEBNext® Ultra RNA Library Prep Kit for Illumina® (NEB England BioLabs, E7530L). The fragmented and randomly primed 2 × 150-bp paired-end libraries were sequenced using an Illumina HiSeq X Ten. The generated sequencing reads were mapped against human genome hg38 using HISAT2 alignment software tools. The read count and FPKM value of each gene was calculated and normalized with StringTie software.

### Detection of chromosomal duplications using eSNP-Karyotyping

eSNP-karyotyping was performed with R packages eSNP-Karyotyping (https://github.com/BenvenLab/eSNPKaryotyping)^30^. BAM files were edited using Picard tools and SNPs were called using the GATK HaplotypeCaller^67^. The SNPs were filtered according to the reading depth and allelic frequency to reduce errors and noise. SNPs with low coverage (below 20 reads) or with low minor allele frequency in the total allele poll (lower than 0.25) were discarded. Next, for each SNP, the major-to-minor frequency ratio was calculated and the table was sorted by the chromosomal position. For visualization, moving medians of the major-to-minor ratios were plotted along the moving medians of the chromosomal positions. Usually, a window of 151 SNPs was used. The *p*-value was calculated with a one-tailed *t*-test comparing the SNP’s major/minor values in the window to the total SNP pool and correcting for multiple testing using FDR correction. In specific cases, to reduce noise, the list of SNPs was further filtered to contain only known SNPs, and SNPs were mixed in different ratios using the SAMtools view and merge functions (https://github.com/samtools/samtools)^68,69^. To determine the necessary read number, different percentages of reads, from 10% up to 100% were randomly selected and analyzed using eSNP-Karyotyping. The samples selected for this assay cover with more than 50M mapped reads. The entire workflow and visualization of the data were performed using R statistical software (v3.5).

### Detection of LOH using eSNP-Karyotyping

A list of common SNPs (Common SNPs 151) in the human genome was obtained from the dbSNP database (http://genome.ucsc.edu/cgi-bin/). For each common SNP, we first determined whether it was homozygote or heterozygote by checking whether it was detected as a valid SNP in our SNP calling. Next, SNPs that were covered by fewer than 20 reads were discarded. The reading depth for each SNP was determined by the SAMtools depth function. For each chromosome, we calculated the number of homozygote and heterozygote SNPs in blocks of 1.5 Mb and plotted them along the chromosome. The entire workflow and visualization of the data were performed using R. To obtain *p*-value, we determined the ratio of the number of homozygotes to heterozygote SNPs for each chromosome arm. Then, we determined each arm if this ratio is statistically different from the rest of the chromosome arms by t-test. The *p*-value list was corrected for multiple testing using FDR correction. True LOH is considered as an arm with *p*-value lower than 0.001 and a homozygote to heterozygote SNPs ratio five times greater than the ratio of all the autosomal chromosomes.

### TP53 mutation genomic sequence

To identify TP53 mutations in MSCs and DOPA-MSCs, we analyzed RNA-seq data of MSCs and DOPA-MSCs. Following sequence alignment to the hg38 human reference genome with HISAT2, single nucleotides divergent from the reference genome were identified using GATK HaplotypeCaller^67^. As sufficient sequencing depth is required to deduce sequence mutation, a threshold of 25 reads per nucleotide was set. The resulting wig files were then plotted using Integrative Genomics Viewer (IGV). The four damaging and high impact mutations (hg19: chr17:7577121:G:C, chr17:7577539:G:A, chr17:7578406:C:T, chr17:7577548:C:T relative to hg38:chr17: 7673803:G:C, chr17:7674221:G:A, chr17:7674229:C:T, chr17:7675088:C:T)^28^ of TP53 were analyzed.

### PD animal models

The adult mice (6–8 weeks, female, 21–24 g), Sprague Dawley rats (8–10 weeks, male, 180–230 g) and rhesus monkeys (male, 8–15 years old, 10–12 kg) used in this study were obtained from Beijing Vital River Laboratory Animal Technology Co., Ltd. (mice), Chengdu Dossy Experimental Animals Co., Ltd. (rats) and Kunming Biomed International (monkeys).

To comprehensively evaluate the efficacy of the DOPA-MSC therapeutic strategy, three approaches were used to create hemi- or bilateral parkinsonism in monkeys. (1) Putamen MPP + (hemiparkinsonism, *n* = 6): The MPP + (a toxic metabolite of MPTP) solution was directly injected into the unilateral putamen, a nucleus of the striatum, with a single dose (0.2 mg/kg) under the guidance of magnetic resonance imaging (MRI) of the monkey. (2) Carotid MPTP (hemiparkinsonism, *n* = 3): The unilateral internal carotid artery and external carotid artery were isolated after the common carotid artery bifurcation was exposed. The origin of the external carotid artery was temporarily clamped. The MPTP solution was slowly injected into the carotid artery (1.5 mg/kg). An extra dose (1.0 mg/kg) was added once the monkey model did not reach the criteria of hemiparkinsonism based on behavioral assays. (3) Bi-ventricle MPP+ (bilateral parkinsonism, *n* = 3): The localization tubes targeting the bilateral ventricles were implanted into the skull of the monkey under MRI guidance. The MPP+ solution (100 μg/each side of the ventricle) was injected twice a week by bilateral localization tubes until we observed persistent parkinsonian symptoms by behavioral assays. For all monkey models, stable hemi- or bilateral parkinsonian symptoms were observed for more than 12 weeks for acute models and 12–18 months for chronic models before an animal was used for the experiments.

Hemiparkinsonian rat models were also used to evaluate the efficacy of DOPA-MSCs in this study. The rat received stereotaxic unilateral injections of 6-OHDA solution (25 µg/150 g, dissolved in saline with 0.2% ascorbic acid) into the right nigra-striatal pathway ([AP] = −4.4 mm, lateral [L] = −1.2 mm, and vertical [V] = −8.2 mm). Before transplantation, these hemiparkinsonian phenotypes were stabilized for three months, and the rats that showed apomorphine-induced rotation >6 rotations per min were selected for further experiments^23,70,71^.

### Cell transplantation

#### Mice

To assess the long-term survival and impact of grafted cells, we transplanted DOPA-MSCs into the striatum of NOD. CB17-Prkdc^scid^/NcrCrl immunodeficient mice (*n* = 8). The DOPA-MSC suspension was prepared at 1 × 10^5^ cells per μl. The mice were anesthetized with 1.25% avertin (2,2,2-tribromoethanol) (intraperitoneally 10 ml/kg), and a total of 1.5 × 10^5^ DOPA-MSCs (1.5 μl suspension) were injected into the striatum at the following coordinates (anterior-posterior [AP] = 0.5 mm, lateral [L] = −2 mm, and vertical [V] = −3.5 mm).

#### Rhesus monkeys

The DOPA-MSC sphere suspension was prepared at 1 × 10^5^ cells per μl. For all monkey models, the cell suspension was injected along four tracts per side (two tracts in the caudate and two tracts in the putamen), three injection sites were in each tract, and 5 μl of sphere suspension was in each injection site (6.0 × 10^6^ in total per side). The coordinates of the targets were obtained using MRI. The DOPA-MSCs were stereotactically transplanted into the lesion side of putamen MPP + and carotid MPTP monkeys (hemiparkinsonism) and bilateral sides of bi-ventricle MPP + monkeys (bilateral parkinsonism). After surgery, monkeys were given antibiotics for 3 days, but no immunosuppressant was given to the monkeys before and after cell transplantation.

#### Rats

The DOPA-MSC suspension was prepared at 1 × 10^5^ cells per μl. The hemiparkinsonian rats randomly received transplantation of 4 μl of DOPA-MSCs (*n* = 13), MSCs (*n* = 3), or PBS (*n* = 3). The coordinates of the injection site were 2 mm posterior, 2.2 mm lateral, and 5.5 mm vertical. No immunosuppressant was given to the rats before or after cell transplantation.

### Study approval

All rodent and monkey procedures were performed following the Laboratory Animal Guideline for Ethical Review of Animal Welfare (GB/T 35892-2018). All procedures were approved in advance by the Laboratory Animal Ethics Committee of Kunming University of Science and Technology (PZWH(Dian)K2021-0014) and the Institutional Animal Care and Use Committee (LPBR202101024).

### Behavioral assays

For all monkey behavioral tests, the behavioral paradigms were performed and observed by three trained technicians independently, who were blinded to the treatments of the monkeys. Another two trained technicians performed all rat behavioral tests.

#### Monkey PD scores

High-resolution video equipment (HDR-XR260, SONY) was used to record all behaviors of monkeys in their living cage. In reference to the Kurlan Scale^72^, the following items were assessed and scored: gait (0–5), tremor (0–5), body posture (0–5), overall motor function (0–5), bradykinesia (0–5), balance coordination (0–5), defensive response (0–5). A score of zero indicates a normal monkey and a maximum score of 35 indicates an animal with severe PD symptoms.

#### Locomotor activities

The locomotor activity of each monkey was analyzed by a commercial primate behavior analysis system ((PrimateScan1.0, CleverSys Inc., USA). In brief, the monkey was placed in a transparent cage (1.2 × 1.0 × 1.0 m) in a quiet room for video recording before and after 6-OHDA treatment. Records and results are stored and analyzed by PrimateScan software (PrimateScan1.0, CleverSys Inc, USA). PrimateScan can automatically identify important body parts of monkeys and record behaviors, including Come Down, Grooming, Jump, Move Left, Move Right, Pace, Sit, Stand Up, Turn, Twitch, Crouch, Hang, Land, etc. The locomotor activities of the monkeys were collected and analyzed with PrimateScan1.0 software.

#### Fine motor skills

A rotating Brinkman board (20 cm in diameter, clockwise at 20 rpm) containing 32 oval slots (15 × 8 × 6 mm, length × width × depth) was deployed to measure manual dexterity^73^. Eight peanuts with a round shape of about 4 mm were placed in the eight holes in the second row of the turntable as rewards. The monkeys were allowed to retrieve peanuts from the slots. The number of rewards successfully retrieved and the peanuts holding time of successful take-ups (measuring frame by frame of the video sequence) were scored.

#### Object discrimination learning

Object discrimination learning test was performed using a 90 cm board with five well with lids of different colors. In the training session, there was only one well covered with a pre-set color (i.e., red) lid on the presentation board, and the monkey was trained to remove the lid of the well and take out the reward (peanut or candy) from the well. Two hours later, in the test session, there were 5 wells covered with different colors on the presentation board, the well with pre-set color (red) lid contained a reward. Monkeys could choose one of the 5 wells to remove the lid to check for candy reward in the chosen boxes in one trial. The position of the pre-set color well switched randomly for each trial during the test. The success rate in 10 consecutive trials was counted.

#### Human intruder test

The human intruder test is a well-established paradigm to measure behavioral response to emotionality, anxiety, or defensive/aggressive situation in captive rhesus macaques^74^. The tested monkey was first placed alone in an isolated cage (1.2 × 1.0 × 1.0 m) by itself in a quiet room for the first 9 min. Then, for the next 9 min, a human entered the room and presented her profile to the animal, standing 2.5 m from the cage while avoiding any eye contact with the animal. The human then left the room for 3 min, reentered the room, and for 9 min remained motionless 2.5 m from the cage while staring, with a neutral face, directly at the animal. Behaviors and emotional responses (screaming, staring, baring teeth, smacking mouth, dodging, circling, jumping, and shaking the cage) were recorded and analyzed^75^.

#### Rotation test

Apomorphine-induced rotations in hemiparkinsonian rats were carried out before cell transplantation and 1, 2, 3, 4, 5, 6, 7, 8, 9, 10, and 11 weeks after cell transplantation. Rotation was recorded and analyzed 30 mins since intraperitoneal injection of apomorphine (0.5 mg kg^−1^), as previously described^76^. Only full-body turns were counted and presented as the average number of rotations per minute.

#### Rotarod test

An accelerating Rotarod (Rota Rod Touch, Panlab Harvard Apparatus) was used to test motor coordination. All animals were pre-trained for three days in order to reach a stable performance. On Day 1, rats were trained on a rotating rod that accelerated from 2 per minute (rpm) to 20 rpm in a period of 300 s three times. On Day 2, rats were trained on a rod accelerated from 3 to 30 rpm thrice. On Day 3, rats were trained on a rod accelerated from 4 to 40 rpm for a period of 300 s. The test was performed from the fourth day on a rotating rod that accelerated from 4 to 40 rpm in a period of 300 s. The period of time that the rats stayed on the rod was monitored. The average duration from three repeated tests of each animal was used for data analysis.

### SPECT study

The single-photon emission computed tomography (SPECT) imaging using ^99^Tc^m^-TRODAT-1 to the label dopamine transporter (DAT) in vivo was performed to evaluate the number of dopamine terminals. The ^99^Tc^m^-TRODAT-1 was prepared from a lyophilized kit^49^. Oral administration of KCLO4 (400 mg) for thyroid blockade was conducted 0.5 h before intravenously injected with 740 MBq (20 mCi) of 99mTc-TRODAT-1. The brain SPECT commenced 2 h later, using a dual-headed rotating gamma camera (Infinia Hawkey, GE Healthcare Ltd) calibrated with a pixel-matrix of 128 × 128 and an energy window of 140 ± 20% keV. The duration of the SPECT imaging was 1.5 h, and the data were reconstructed by a back-projection algorithm with a Butterworth 3D post-filter and attenuation correction. The regions of interest (ROIs) were manually drawn on the bilateral striatum (STr, including caudate and putamen) and cerebellum (CB, serving as background area) in reference to the CT images. The analysts who drew ROIs manually were blinded to the genotype. The striatal radioactivity uptakes were calculated by subtracting the mean counts per pixel in CB from the mean counts per pixel in STr in each hemisphere, and dividing the result by the mean counts per pixel in the background: (STr-CB)/CB. The striatal radioactivity of each monkey was the average value of 3 consecutive layers. The measurement of SPECT data was performed using eFilm Workstation 3.4 software (Merge Healthcare, Milwaukee, WI, USA).

### Tissue processing

Rats were sacrificed with CO_2_ and immediately perfused with the first phosphate-buffered saline and then 4% PFA in PBS to fix tissues. Brains were extracted, post-fixed in 4% PFA, and soaked in 30% sucrose solutions for 2–5 days. They were sectioned at 50 μm on a Vibrating Microtome (LEICA VT 1200 S) after embedding in agarose.

### Immunofluorescence

Cells were fixed in 4% PFA. For immunostaining, brain sections were randomly chosen based on the anatomical location from different serial collecting wells for an individual rat. Each collecting well contained approximately 6–8 serial sections from anterior to posterior at a spacing of every 300 μm. Blocking solution for fixed cells and brain sections included 10% normal donkey serum (Jackson Immunoresearch) in PBS surplus with 0.4% Triton X-100. Primary antibodies (Tyrosine Hydroxylase: millipore; DDC Antibody: Novus Biologicals; Anti-GCH1 Antibody: abcam; Mouse anti-human nuclei monoclonal antibody: Millipore; Ki-67 Monoclonal Antibody: Thermo Fisher Scientific; Anti Iba1: Wako; Anti-CD45: Bioss; Anti-Glial Fibrillary Acidic Protein Antibody: millipore) were diluted in PBS containing 2.5% normal donkey serum and 0.4% Triton X-100 and incubated overnight at 4 °C according to manufacturer recommendations. Secondary antibodies conjugated to Alexa488, Alexa555, or Alexa647 (Molecular Probes) diluted in PBS containing 5% normal donkey serum were incubated for 2 h at room temperature. Nuclear counterstain was visualized with 4′,6-diamidino-2-phenylindole (DAPI, Thermo Fisher). After PBS wash, stained sections were mounted on adhesion microscope glass slides (MXB Biotechnologies).

### Collection of cerebrospinal fluid

The cerebrospinal fluid (CSF) of monkeys was sampled by lumbar puncture in L3-4 interspace when the monkeys were under anesthetized by intramuscular injection of atropine (0.05 mg/kg) and ketamine (10 mg/kg). The CSF was protected from light during the sampling process, then the CSF sample (0.8 ml) was centrifuged at 8000 × *g* for 15 min at 4 °C and immediately frozen at −80 °C until analysis. CSF of rats samples is taken from the cisterna magna using a method as described previously^77^. Dopamine concentration of CSF assayed by dopamine ELISA Kit (Abnova, Catalog Number KA1887).

### Statistical analyses

All experiments and data analyses were conducted blind to experimental groups. No statistical methods were used to pre-determine sample sizes. All data analyses in monkey experiments were present as mean ± s.d. Two-way analysis of variance (ANOVA) with Dunnett’s multiple comparisons test was performed. *P < 0.05; **P < 0.01; ***P < 0.001; Significance before and after transplantation was assessed by a two-tailed Student’s *t*-test. Two-tailed Pearson’s correlation analysis was performed, and r and *P* values are shown. All data analyses in rat experiments were presented as mean ± standard error. One-way AONVA analysis of variance was used to compare the differences between groups, and the Bonferroni test was used for evaluating the significance difference. In the two rat behavioral tests, Two-way ANOVA followed by the Holm–Sidak test was used for evaluating the significance difference. In all statistical analyses, *p* < 0.05 was considered significant.

## Supplementary information

Supplemental files

Video S1. Changes of PD symptoms in the Putamen MPP+-2 monkey before and after DOPA-MSC transplantation, relative to Figure 3B.

Video S2. Changes of PD symptoms in the Carotid MPTP-2 monkey before and after DOPA-MSC transplantation, relative to Figure 3D

Video S3. The speed and correct times of finger feedings for the Bi-ventricle MPP+-2 monkey were markedly improved after DOPA-MSC transplantation by FMS assay.

Video S4. Changes of PD symptoms in the Bi-ventricle MPP+2 monkey before and after DOPA-MSC transplantation, relative to Figure 4A.

Video S5. Cognitive test of PD monkey before and after DOPA-MSC transplantation, relative to Figure 4E.

Video S6. Emotional response test of the Bi-ventricle MPP+-1 monkey, relative to Figure 4F.

## Data Availability

The raw sequence data reported in this paper have been deposited in the Genome Sequence Archive (Genomics, Proteomics & Bioinformatics 2021) at National Genomics Data Center (Nucleic Acids Res 2022), China National Center for Bioinformation/Beijing Institute of Genomics, Chinese Academy of Sciences (GSA-Human: HRA003465) that are publicly accessible at https://ngdc.cncb.ac.cn/gsa-human. Other data generated and/or analyzed during the current study are included in this published article (and its Supplementary Information files).
