# Supplementary material for: Genetically engineered mesenchymal stem cells with dopamine synthesis for Parkinson’s disease in animal models
Source: NPJ Parkinsons Dis. 2022 Dec 22;8:175. doi: 10.1038/s41531-022-00440-6 (PMC9780305; doi:10.1038/s41531-022-00440-6)
Supplement: Supplementary file 1 — Supplemental files [file 41531_2022_440_MOESM1_ESM.pdf]

Supplemental Figures and table

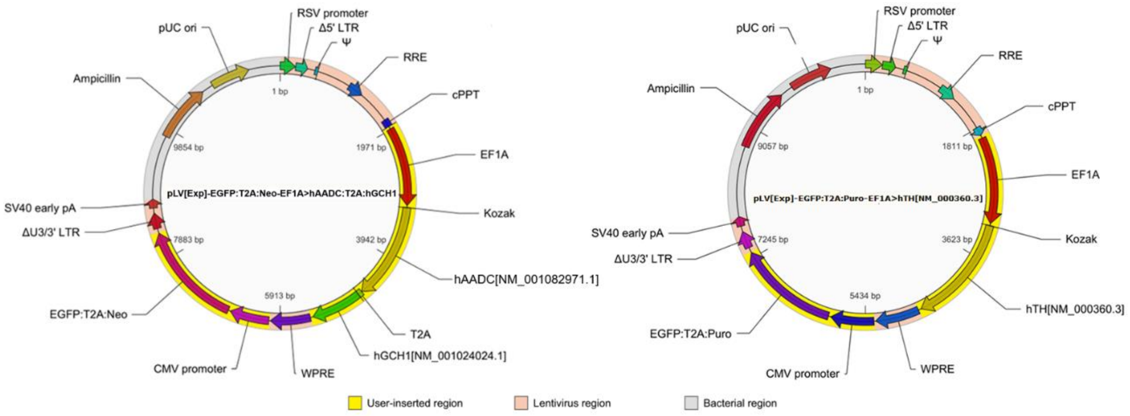

Supplementary Figure 1. The plasmid vector map of lentivirus carrying human CDS of *TH*, *ADDC*, and *GCH1*, relative to Figure 1.

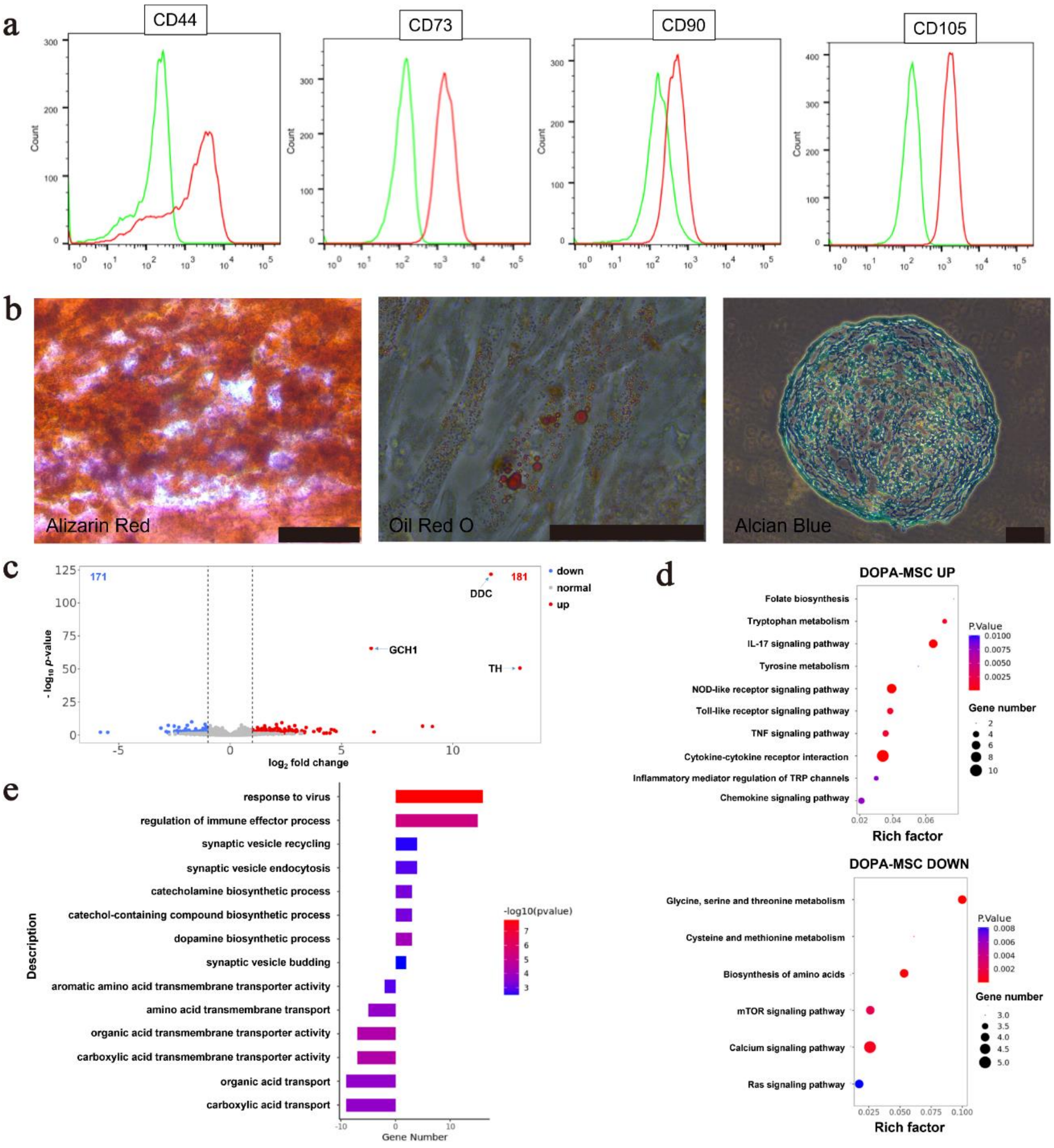

Supplementary Figure 2. Identification and transcriptome analysis of DOPA-MSCs, relative to Figure 1

a. The expressions of the MSC markers CD44, CD73, CD90 and CD105 in DOPA-MSCs were analyzed by flow cytometry. Green represents negative Isotype Control and red represent positive result of the DOPA-MSCs

b. Differentiation properties of DOPA-MSCs. The differentiated osteogenic, adipogenic, and chondrogenic lineages were evaluated by alizarin red staining (Scale bar:100 μm), oil red O staining (Scale bar:100 μm), and alcian blue staining (Scale bar:250 μm), respectively.

c. Volcano plots demonstrating differentially expressed genes (DEGs) ($\log_2$ (fold change) > 1 or < -1 and p-value < 0.05) between DOPA-MSCs and MSCs. Arrows indicate the interest genes that are upregulated or downregulated in DOPA-MSCs.

d. The Kyoto Encyclopedia of Genes and Genomes (KEGG) pathway analysis of upregulated or downregulated genes in DOPA-MSCs compared with in MSCs. The KEGG pathways were analyzed by the web-based gene enrichment analysis tool DAVID. The size of the circle represents the number of genes.

e. The gene ontology (GO) analysis of upregulated or downregulated genes in DOPA-MSCs compared with in MSCs. The GO terms were analyzed by the web-based gene enrichment analysis tool DAVID.

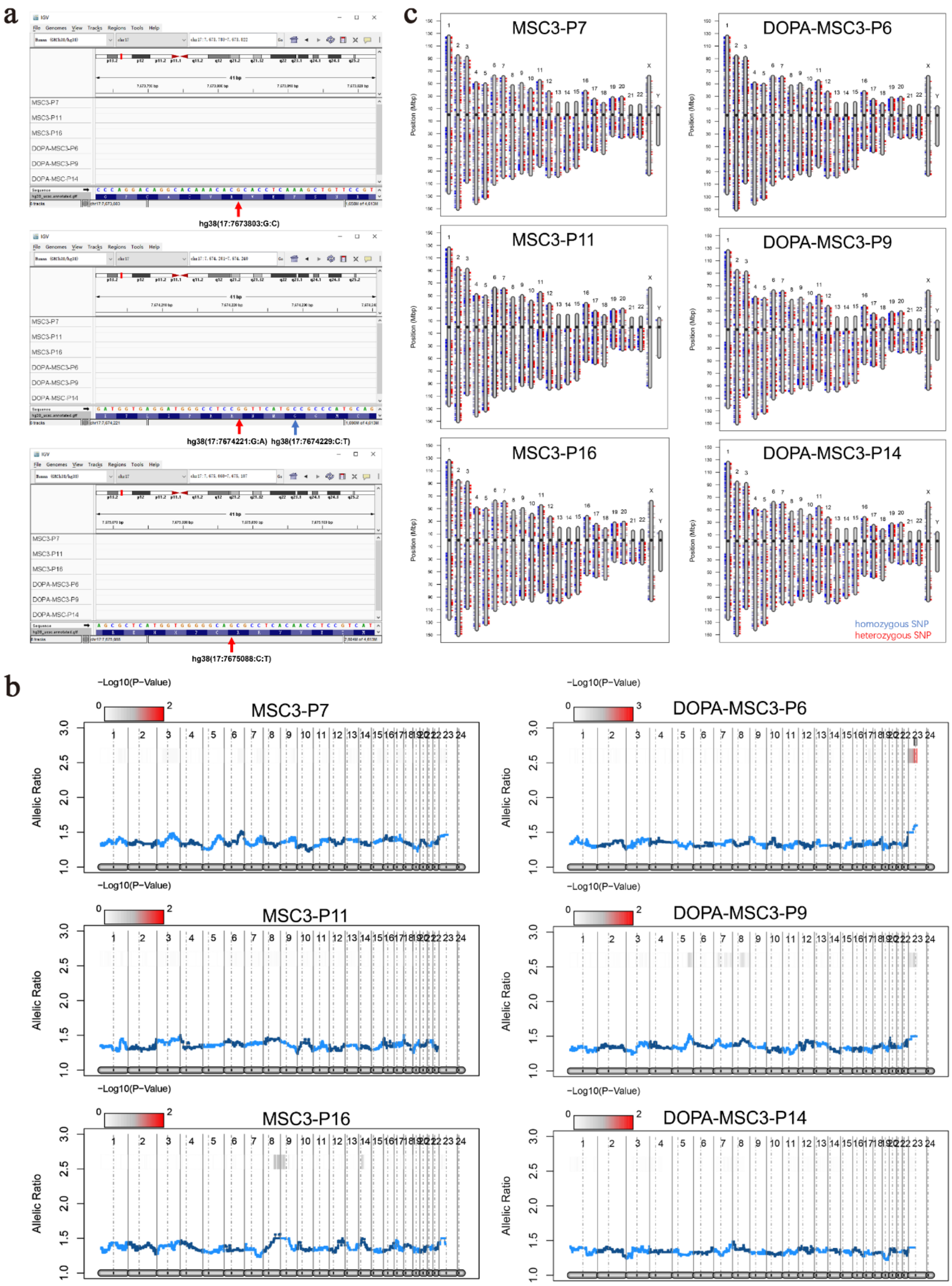

Supplementary Figure 3. Transcriptome analysis of genomic stability, relative to Figure 1.

a. Analysis of cancer-related TP53 mutations using RNA-Seq data from MSCs and DOPA-MSCs. Arrows indicate no reported mutation site of TP53 were detected by analyzing the RNA-seq data.

b. eSNP-Karyotype analyses of MSCs and DOPA-MSCs. Color bars represent the FDR-corrected p-value. Positions with a p-value lower than 0.01 are marked by a black line.

c. LOH detection of MSCs and DOPA-MSCs using eSNP-Karyotyping. Blue lines represent expressed homozygous SNPs and red lines represent expressed heterozygous SNPs. Color intensity represents the SNP density within a specific region. Regions of statistically significant LOH are highlighted with a yellow background.

eSNP-Karyotyping and LOH analysis from b and c showed that there is neither chromosomal aberrations nor loss of heterozygosity (LOH) events detected in DOPA-MSCs according to reference 30.

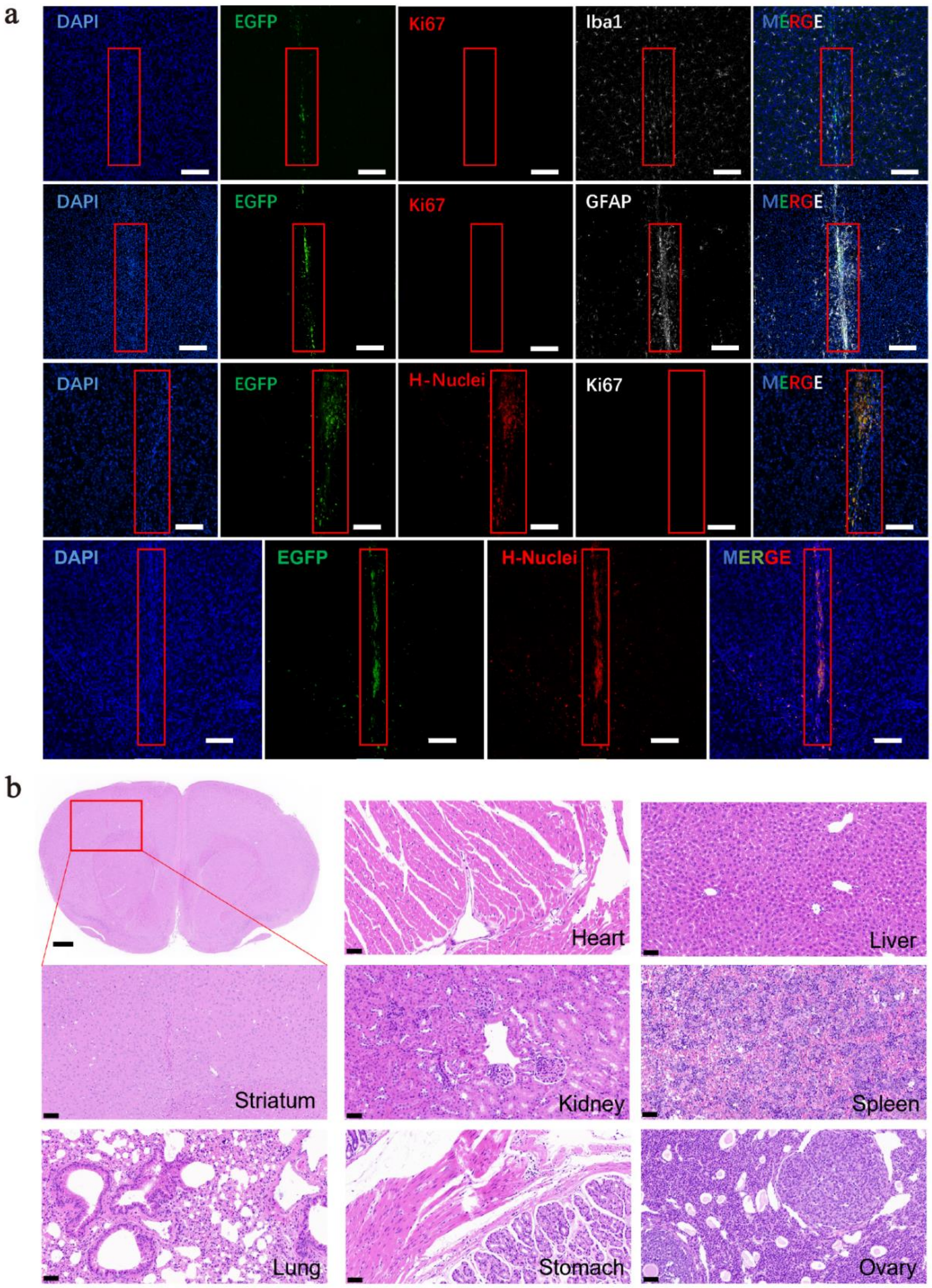

Supplementary Figure 4. Long-term survival of grafted cells and histopathological assessments of the major organs in mice 6 months after DOPA-MSC grafts

8 mice were used in the experiment. All animals exhibited consensus results. The representative figures were shown.

a. Absence of tumor formation and long-term survival of grafted DOPA-MSCs in the striatum of NOD.CB17-Prkdc^{scid}/NcrCrl mice. Scale bars:100 µm.

b. Histopathological assessments of the major organs in mice on Day 150 post grafts. Scale bars: top left panel, 500 µm; other panels, 50 µm.

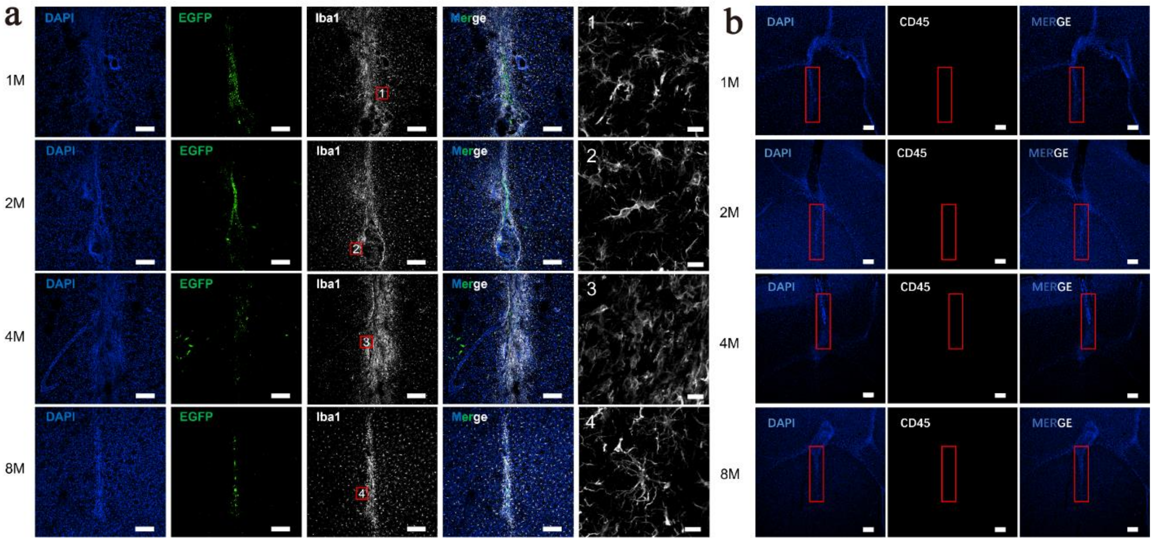

Supplementary Figure 5. Immune response evaluation in PD rat brains after transplantation of DOPA-MSCs

a. The immunostaining of microglial marker Iba1 for PD rat brains at indicated time following DOPA-MSC grafts. No immunosuppressive drug was used after cell transplantation. DOPA-MSCs were labeled by EGFP signals. Scale bars:200 μm; 10 μm in the zoom panel. Right panels indicate enlarged relative to the left remarked rectangle area.

b. The immunostaining of microglial marker CD45 for PD rat brains at indicated time following DOPA-MSC grafts. No immunosuppressive drug was used after cell transplantation. Scale bars:200 μm.

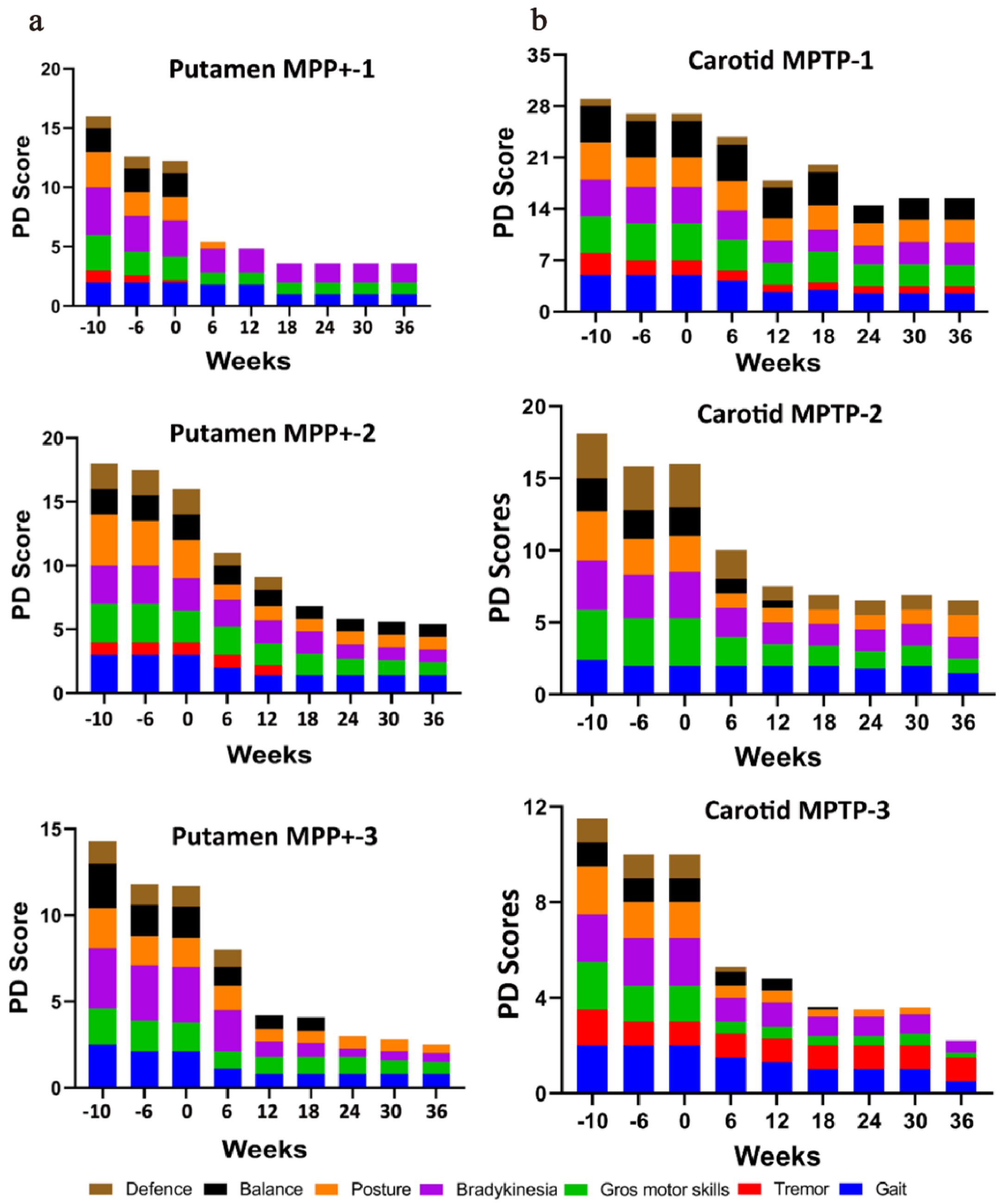

Supplementary Figure 6. Details of PD scores in putamen MPP+-lesioned and carotid MPTP-lesioned PD monkeys, respectively, relative to Figure 3.

a. Each behavior items of PD symptoms in the Putamen MPP+-1, Putamen MPP+-2, Putamen MPP+-3 monkeys induced by administration of MPP+ into the putamen before and after transplantation of DOPA-MSCs.

b. Each behavior items of PD symptoms in the Carotid MPTP-1, Carotid MPTP-2, Carotid MPTP-3 monkeys induced by carotid infusion of MPTP before and after transplantation of DOPA-MSCs.

In A and B, each value is from the average result of 6 independent tests.

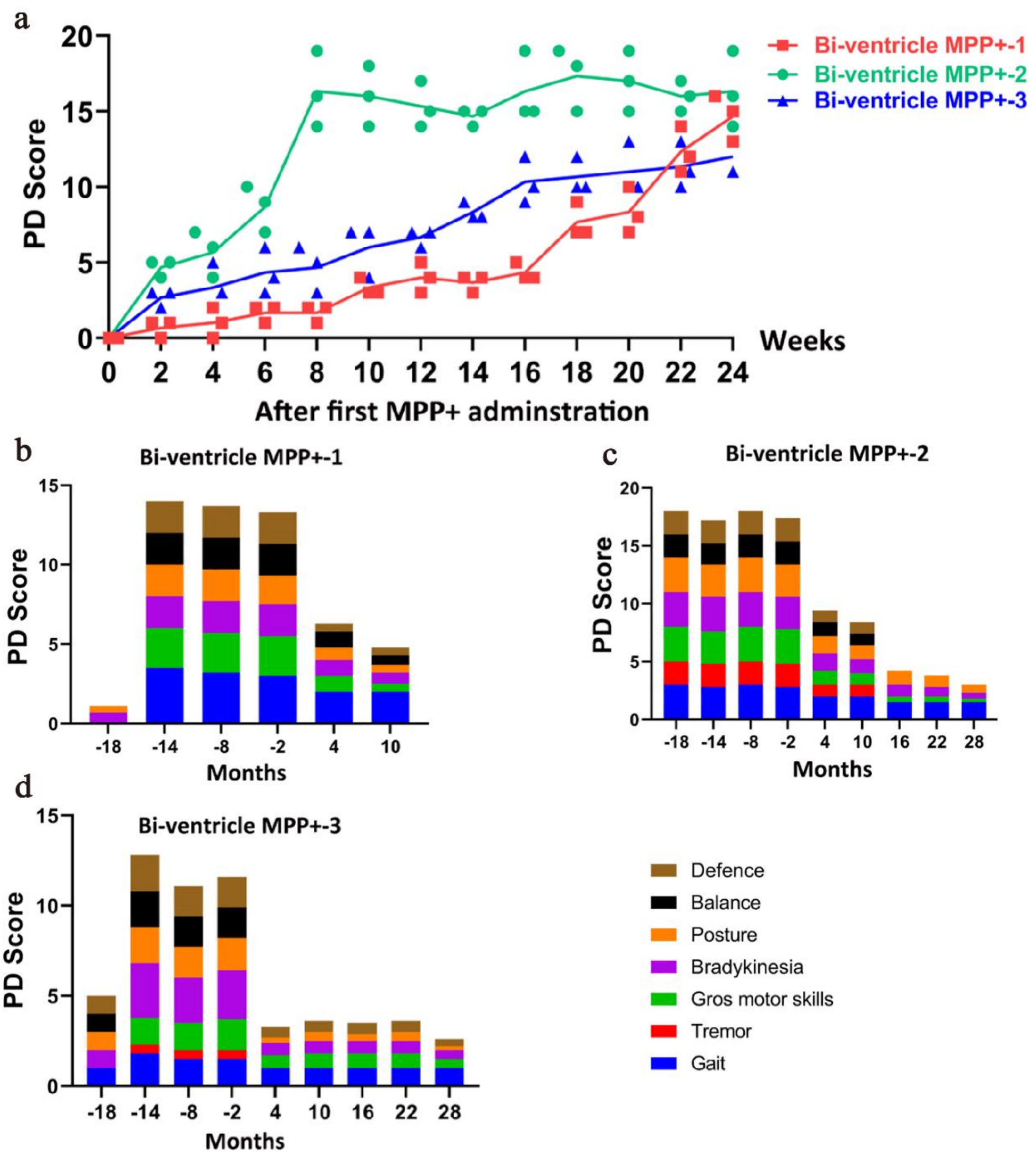

Supplementary Figure 7. Details of PD scores in bilateral parkinsonian chronic monkeys induced by lateral ventricular administration of MPP$^+$, relative to Figure 4.

a. Bilateral parkinsonian chronic monkeys were chronically induced by lateral ventricular administration of MPP$^+$.

b-d. Each behavior items of PD symptoms in the Bi-ventricle MPP+-1 (**b**), Bi-ventricle MPP+-2 (**c**), Bi-ventricle MPP+-3 (**d**) monkeys before and after transplantation of DOPA-MSCs. Each value is from the average result of 6 independent tests.

Supplementary table 1. Information of rhesus monkey used in the study

Animal	Gender	Age	Lesion method	Duration/ months	Transplanted cells	Grafting region
Putamen MPP+-1	Male	8	MPP+/PU (Putamen)	3	1*10^7 DOPA-MSCs	Putamen+caudate
Putamen MPP+-2	Male	10	MPP+/PU (Putamen)	3	1*10^7 DOPA-MSCs	Putamen+caudate
Putamen MPP+-3	Male	10	MPP+/PU (Putamen)	3	1*10^7 DOPA-MSCs	Putamen+caudate
Putamen MPP+-4	Male	11	MPP+/PU (Putamen)	3	1*10^7 MSCs	Putamen+caudate
Putamen MPP+-5	Male	9	MPP+/PU (Putamen)	3	1*10^7 MSCs	Putamen+caudate
Putamen MPP+-6	Male	12	MPP+/PU (Putamen)	3	1*10^7 MSCs	Putamen+caudate
Carotid MPTP-1	Male	9	MPTP/CA (Carotid artery)	3	1*10^7 DOPA-MSCs	Putamen+caudate
Carotid MPTP-2	Male	15	MPTP/CA (Carotid artery)	3	1*10^7 DOPA-MSCs	Putamen+caudate
Carotid MPTP-3	Male	11	MPTP/CA (Carotid artery)	3	1*10^7 DOPA-MSCs	Putamen+caudate
Bi-ventricle MPP+-1	Male	11	MPP+/LV (lateral Ventricle)	18	6*10^6 DOPA-MSCs	Putamen+caudate
Bi-ventricle MPP+-2	Male	10	MPP+/LV (lateral Ventricle)	18	6*10^6 DOPA-MSCs	Putamen+caudate
Bi-ventricle MPP+-3	Male	15	MPP+/LV (lateral Ventricle)	18	6*10^6 DOPA-MSCs	Putamen+caudate
